# Activation and Autoinhibition Mechanisms of NLR Immune Receptor Pi36 in Rice

**DOI:** 10.3390/ijms25137301

**Published:** 2024-07-02

**Authors:** Yang Yang, Liu Tan, Xingzhe Xu, Qiaoyi Tang, Ji Wang, Shiyue Xing, Rui Wang, Ting Zou, Shiquan Wang, Jun Zhu, Shuangcheng Li, Yueyang Liang, Qiming Deng, Ping Li

**Affiliations:** 1 State Key Laboratory of Hybrid Rice, Rice Research Institute, Sichuan Agricultural University, Chengdu 611130, China; sichuantufei@sina.cn (Y.Y.); tanliu20010418@163.com (L.T.); xxz07012220@163.com (X.X.); 18113705376@163.com (S.X.); sqwangscau@163.com (S.W.); zhujun987@126.com (J.Z.); 2 State Key Laboratory of Crop Gene Exploration and Utilization in Southwest China, Sichuan Agricultural University, Chengdu 611130, China; tangqy04675@163.com (Q.T.); m18190518112@163.com (J.W.); 15775891185@163.com (R.W.); lisc926105@163.com (S.L.); liangyueyang1984@hotmail.com (Y.L.)

**Keywords:** Pi36, *Nicotiana benthamiana*, cell death, self-association

## Abstract

Nucleotide-binding and leucine-rich repeat receptors (NLRs) are the most important and largest class of immune receptors in plants. The *Pi36* gene encodes a canonical CC-NBS-LRR protein that confers resistance to rice blast fungal infections. Here, we show that the CC domain of Pi36 plays a role in cell death induction. Furthermore, self-association is required for the CC domain-mediated cell death, and the self-association ability is correlated with the cell death level. In addition, the NB-ARC domain may suppress the activity of the CC domain through intramolecular interaction. The mutations D440G next to the RNBS-D motif and D503V in the MHD motif autoactivated Pi36, but the mutation K212 in the P-loop motif inhibited this autoactivation, indicating that nucleotide binding of the NB-ARC domain is essential for Pi36 activation. We also found that the LRR domain is required for D503V- and D440G-mediated Pi36 autoactivation. Interestingly, several mutations in the CC domain compromised the CC domain-mediated cell death without affecting the D440G- or D503V-mediated Pi36 autoactivation. The autoactivate Pi36 variants exhibited stronger self-associations than the inactive variants. Taken together, we speculated that the CC domain of Pi36 executes cell death activities, whereas the NB-ARC domain suppressed CC-mediated cell death via intermolecular interaction. The NB-ARC domain releases its suppression of the CC domain and strengthens the self-association of Pi36 to support the CC domain, possibly through nucleotide exchange.

## 1. Introduction

Plant canonical NLRs (nucleotide-binding leucine-rich repeat receptors) typically contain a central nucleotide-binding and oligomerization domain (NOD), a C-terminal leucine-rich repeat (LRR) domain, and a variable N-terminal domain [1]. NLR activation triggers immune responses, accompanied by a rapid programmed cell death response, a type of programmed cell death that restricts pathogens to the infection site [2].

NLRs can be classified into three main groups based on their variable N-terminal domains, namely, coiled-coil (CC), toll/interleukin-1 receptor-like (TIR), and RPW8-like CC (CC_R_) [3]. The N-terminal domains are generally considered signaling components, and their overexpression sometimes triggers cell death in *N. benthamiana* [4]. In CC-NLRs, some CC domains of NLR proteins such as MLA10, Sr33, Sr50, Rp1-D21, Pvr4, and ZAR1 were reported to induce cell death in *N. benthamiana* [5,6,7,8,9]. However, the CC domains of RPM1, Rx, Sw-5, Sr35, and RPS5 could not induce cell death [10]. NOD, also referred to as NB-ARC, acts as a molecular switch that regulates NLR activity by binding and hydrolyzing nucleotides. The ADP- and ATP-bound forms of the NB-ARC domain are associated with the “off” and “on” states of NLRs, respectively [1,11,12]. The NB-ARC domain comprises the NB domain and the ARC domain, with the ARC domain further divided into HD1 and WHD subunits [13]. The LRR domain plays a dual role in autoinhibition and effector perception in plant NLRs. Deletion or domain swaps of LRR domains led to constitutive activation in some plant NLRs such as RPS5, MLA10, Pvr4, RPP1A, and L6 [6,9,14,15,16]. The LRR domain can recognize effectors through direct interaction with effectors or through interaction with guardees, which are targeted by effectors [13,14,17,18,19,20].

In general, plant NLRs are present in an inactive state in the absence of corresponding effectors, primarily due to intramolecular interactions among their domains [21,22]. The interactions between the C-terminal part of the NB-ARC domain and the N-terminal part of the LRR domain [7,14,23,24,25,26,27], and between the N-terminal domains and the NB-ARC domain [7,14,24,28], are particularly crucial for NLR autoinhibition, which explains the autoactivation conferred by mutation in these interaction regions and inappropriate domain or region combinations/swapping. In addition, the intramolecular interactions mediated by NLR autoinhibition are further stabilized by ADP binding [13]. The bound ADP forms a conserved set of interactions within the NB-ARC domain, including interactions with the P-loop and MHD motif via its β- and γ-phosphate groups, respectively [13,21,29,30]. Strongly supporting the role of ADP binding in NLR autoinhibition, disrupting the interaction between ADP and the MHD motif in the ARC subdomain, leads to constitutive activation in a large number of plant NLRs [7,16,31,32,33,34].

The ADP/ATP exchange mainly achieved by the NB-ARC domain is crucial for immune function mediated by NLRs. The importance of the ADP/ATP exchange is demonstrated by several functional mutants yielded by mutations in or next to the motifs of the NB-ARC domain in different plant NLRs [25,28,29,30,31]. For example, the P-loop and RNBS-B motifs form extensive interactions with the β-phosphate and γ-phosphates of ATP, respectively, while mutations in the motif usually lead to the loss of function in many plant NLRs due to impairing the ability of ATP binding [1,12,18,19,35,36,37,38,39]. The MHD motif binds the β-phosphate of ADP. Mutations affecting the histidine or aspartate of the MHD motif frequently lead to the constitutive activation of plant NLRs [6,16,31,32]. In some cases, mutations in the RNBS-A and Walker B motif with impaired ATP hydrolysis activity can confer autoactivity [12,23,40,41]. Oligomerization/self-association is considered as a common mechanism in NLR activation [21]. Recently, the structures of pentameric or tetrameric complexes formed by oligomeric ZAR1, Sr35, RPP1, and Roq1 have been reported to be necessary for cell death and resistance activities in these NLRs [17,18,19,37]. Consistent with this notion, the N-terminal domains of several plant NLRs can also form self-association, which is necessary for their cell death activity, further supporting that oligomerization plays an important role in NLR activation [5,28,42,43,44]. Oligomerization of NLRs may induce the close proximity of the N-terminal signaling domains to form high-level functional structures, enabling their scaffolding, channel, or NADase holoenzyme [30]. N, RPP1, ZAR1, and Tm-2^2^ form self-association upon specific recognition of their corresponding effectors [13,28,37,45,46], but RPS5, RPM1, Rp1, and MLA1 can self-associate at both pre-activation and post-activation [7,23,25,42]. In addition to ADP/ATP exchange and oligomerization/self-association, sequential conformational changes are involved in NLR activation [30,47]. Upon effector recognition, a steric clash occurs in the NB domain and causes dramatic changes or structural remodeling in the NB domain, rotating NB outward for ADP/ATP exchange. Following ATP binding, NLRs undergo additional structural remodeling with the rotation of the WHD-LRR fragment, finally resulting in the assembly of resistosomes and immune function [19,37].

Rice blast, caused by *Magnaporthe oryzae*, is one of the most serious diseases in rice cultivation. The *Pi36* gene encodes a canonical CC-NBS-LRR protein that confers resistance to rice blast fungal infections [48]. To elucidate the activation and autoinhibition mechanisms of Pi36, a series of functional analyses were conducted, with a primary emphasis on elucidating the roles of its domains in cell death induction or modulation. The results revealed that the CC domain functions as signaling domains in Pi36. Pi36 CC-mediated cell death requires self-association, and the self-association ability is correlated with the cell death level. The nucleotide exchange and self-association primarily conferred by the NB-ARC domain are crucial for Pi36 activation. The NB-ARC domain suppresses CC-mediated cell death through intermolecular interaction, while NB-ARC may release the repression of the CC domain through nucleotide exchange and enhanced self-association of Pi36 with the participation of the LRR domain. The enhanced self-association of Pi36 may promote the self-association of the CC domain, which will be beneficial to the CC function.

## 2. Results

### 2.1. The Pi36 CC Domain Induces Cell Death

**Figure 1:**
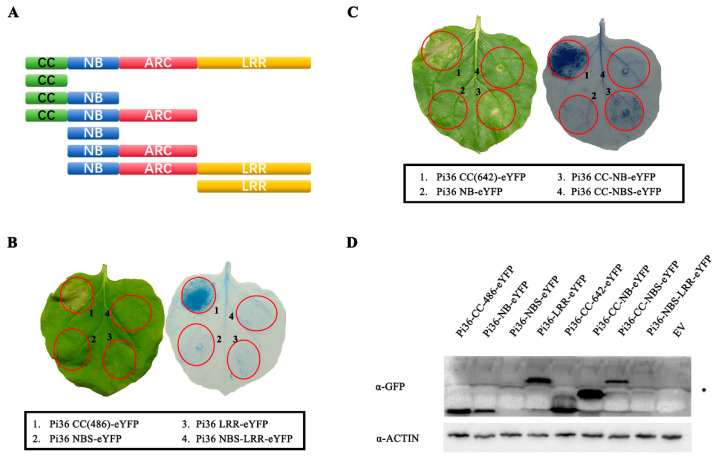
CC domain is the only one that can induce cell death in Pi36. (**A**) Schematic diagram of the Pi36 domain structure and fragments expressed in *N*. *benthamiana.* (**B**,**C**) Cell death phenotype of Pi36 fragments in *N*. *benthamiana*. All proteins were C-terminally tagged with eYFP and transiently expressed in *N*. *benthamiana*. Images were captured at 7 days post-infiltration (dpi), followed by trypan blue staining. The CC domain induced strong cell death, and CC-NB induced weak cell death. (**D**) Protein expression levels of Pi36 fragments shown by Western blot. Total proteins were extracted from *N*. *benthamiana* leaves at 20 h post-infiltration (hpi) and subjected to immunoblotting using anti-GFP and anti-Actin antibodies. The asterisk indicates non-specific bands.

*Pi36* encodes a CC-NBS-LRR protein, highly similar to the MLA family proteins MLA10, Sr33, and Sr35 (Figure S1) [5,48]. To test whether Pi36 functions similarly to the MLA family proteins in cell death induction, a series of Pi36 fragments comprising the CC, NB, NB-ARC, LRR, CC-NB, CC-NB-ARC, and NB-ARC-LRR fragments (Figure 1A) were designed with reference to MLA10 [6]. Subsequently, these fragments were C-terminally tagged with the enhanced yellow fluorescent protein (eYFP) tag, and transiently overexpressed in *N. benthamiana*. As shown in Figure 1B,C, the CC domain (1-162aa or 1-214aa) induced a stronger cell death, whereas the CC-NB fragment induced a weak cell death. Interestingly, Pi36 CC-NB-ARC could not induce cell death when overexpressed in *N. benthamiana*. In contrast, no cell death phenotype was observed upon overexpression of other Pi36 fragments without the CC domain, including the NB, NB-ARC, NB-ARC-LRR, and LRR fragments. Western blotting analysis confirmed the proper expression of all Pi36 fragments (Figure 1D). To eliminate the potential influence from the eYFP tag, these fragments were also expressed without tag fusion in *N. benthamiana*. Consistent with our earlier results, only the CC and CC-NB fragments induced visible cell death (Figure S2). Overall, these results indicate that the CC domain of Pi36 possesses cell death induction ability. Moreover, the cell death activity of the CC domain is negatively regulated by other domains: the NB-ARC domain inhibits the function of the CC domain, whereas the NB domain partially suppresses CC-mediated cell death.

### 2.2. Cell Death Induced by Pi36 CC Domain Is Dependent on Its Self-Association

**Figure 2:**
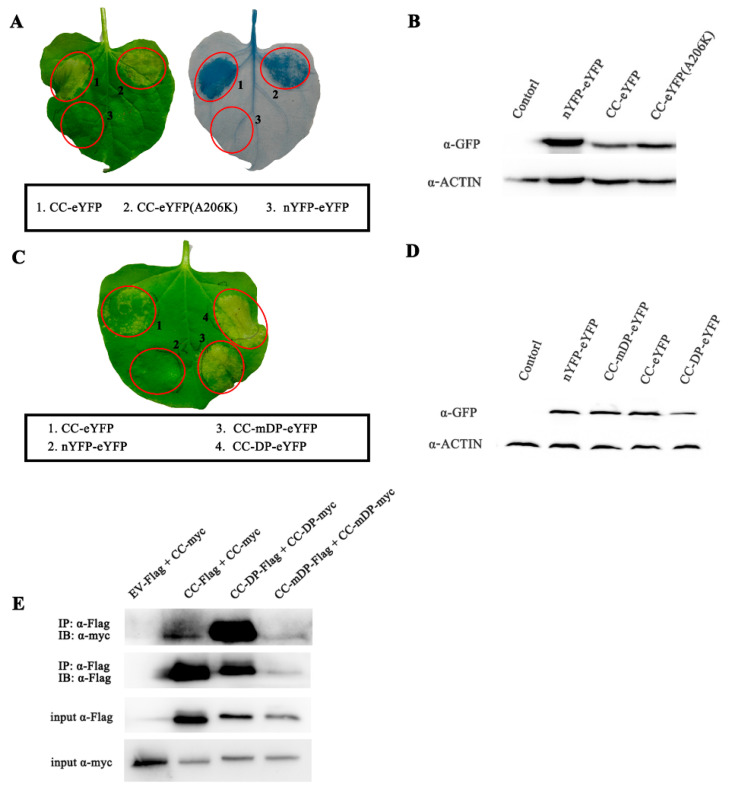
The self-association ability is correlated with the level of cell death mediated by the Pi36 CC domain. (**A**) Cell death mediated by the CC fused with eYFP or monomeric eYFP (carrying the mutation A206K). The monomeric eYFP reduced CC-mediated cell death. (**B**) Protein expression levels of the CC domain fused with eYFP and its mutant. (**C**) Cell death mediated by CC fused with a dimerization peptide (DP) or a mutant DP. The CC domain fused with DP exhibited stronger cell death than CC fused to the mutant DP (mDP). (**D**) Protein expression levels of CC with different peptides. For cell death assay (**A**,**C**), Pi36 CC variants were transiently expressed in *N*. *benthamiana*, and representative pictures were taken at 7 dpi. These experiments were repeated at least three times with similar results. For detection of protein expression levels (**B**,**D**), proteins were extracted from agroinfiltrated leaves at 20 hpi, and detected by Western blot using anti-GFP and anti-Actin antibodies. (**E**) The dimerization peptide (DP) significantly enhanced the self-association of the CC fusion protein. CC wild-type and CC with DP or mDP tagged with Flag and Myc were transiently expressed in the indicated combinations. Proteins extracted from leaves at 20 hpi were subjected to immunoprecipitation with anti-Flag beads, followed by immunoblotting using anti-Myc and anti-Flag antibodies.

To investigate the correlation between self-association and cell death ability in the Pi36 CC domain, the self-association strength of the Pi36 CC domain was artificially reduced and enhanced to assess whether it affected the cell death activity. To reduce the self-association, the Pi36 CC domain was fused with eYFP^A206K^. The mutation A206K can disrupt the dimerization of fluorescent proteins; the modified fluorescent proteins were used to reduce the self-association of their fusion proteins [28,49,50,51]. Pi36 CC-eYFP^A206K^ triggered a weaker cell death than that of CC-eYFP, while their protein levels were similar (Figure 2A,B). Furthermore, a 7-aa dimerization peptide (DP, EFLIVKS), which is usually used to enhance protein self-association, was fused to the CC domain of Pi36 [28,52]. Pi36-CC-DP-eYFP and its mutant form, Pi36-CC-mDP-eYFP, were also transiently expressed in *N. benthamiana*. Co-immunoprecipitation (co-IP) assays confirmed that the Pi36 CC domain could self-associate, and the dimerization peptide (DP) significantly enhanced the self-association of the Pi36 CC domain (Figure 2E). The results from the transient expression showed that CC-DP-eYFP induced a stronger cell death compared to CC-mDP-eYFP and CC-WT-eYFP, while the protein levels of CC-DP-eYFP were similar to those of Pi36-WT-eYFP (Figure 2C,D). Taken together, these findings indicate that the self-association ability of the Pi36 CC domain is correlated with the level of cell death, and that dimerization promotes Pi36 CC domain-mediated cell death.

**Figure 3:**
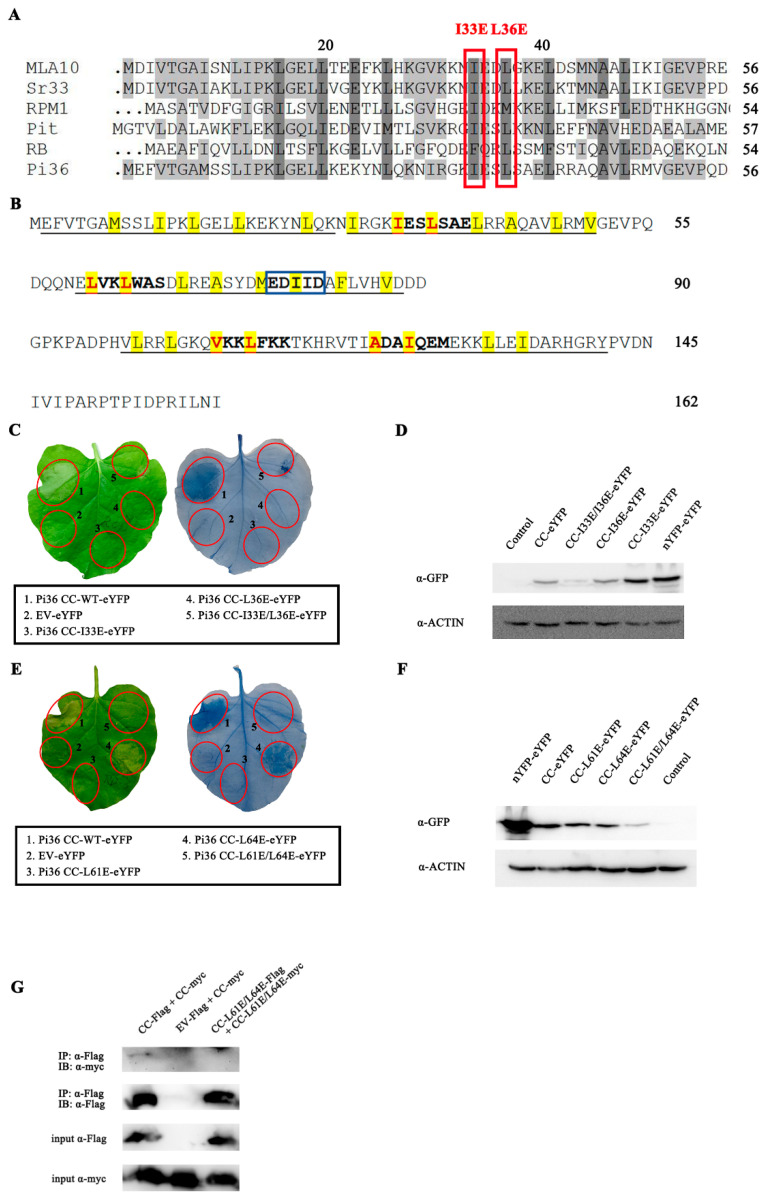
Conserved hydrophobic residues contribute to Pi36 CC-mediated cell death and self-association. (**A**) Multiple alignments of Pi36 with various CNLs in region of N-terminal CC domain. Conserved hydrophobic residues (I33 and L36) were labeled in red boxes. (**B**) The hydrophobic amino acids of heptad repeat within Pi36 CC domains. The hydrophobic residues were highlighted in yellow, with residues selected for mutagenesis analysis marked in red and bold. The EDVID motif was indicated in blue box. (**C**,**E**) Mutations in hydrophobic residues compromised Pi36 CC domain-mediated cell death. C-terminal eYFP-fused CC mutants were transiently expressed in *N*. *benthamiana*. Independent experiment was repeated at least 3 times with similar results, and representative pictures were taken at 7 dpi. (**D**,**F**) Protein expression levels of Pi36 CC mutants by Western blot. Total proteins extracted from agroinfiltrated *N*. *benthamiana* leaves at 20 hpi were subjected to immunoblot analysis using anti-GFP and anti-Actin antibodies. (**G**) Mutations in hydrophobic residues influenced the self-association of the Pi36 CC domain. Self-association of CC domain with double mutation L61E/L64E was compared with wild-type CC domain through co-IP. Indicated combinations of CC fusion proteins were transiently expressed in *N*. *benthamiana*. Proteins collected from leaves at 20 hpi were immunoprecipitated with anti-Flag beads, followed by immunoblotting with anti-Flag and anti-Myc antibodies.

The hydrophobic residues (I31 and L36) are highly conserved among these known NLRs, including Pi36 (Figure 3A), and several heptad repeats were discovered in the Pi36 CC domain (Figure 3B). The heptad repeats are essential for the self-association formation of the CC domain [53,54]. Substitutions of these two conserved hydrophobic residues in the heptad repeats lead to a loss of self-association and/or cell death activity of CC domains in various plant NLRs, such as MLA10, RB, RPM1, and Pit [25,42,55,56]. To test whether the hydrophobic residues influence the cell death activity and self-association of the CC domain, several Pi36 CC domain mutants were generated by converting these residues to negatively charged glutamic acid (Figure 3B) [6]. The single mutations (I31E, L34E, L64E, V107E, A121E, and I124E) and the double mutations (I31E/L34E, L61E/L64E, V107E/L110E, and A121E/I124E) completely compromised the CC domain-mediated cell death (Figure 3 and Figure S3). When the mutations L61E or L110E were introduced into the Pi36 CC domain, the intensity of cell death was reduced compared to the wild-type Pi36 CC domain (Figure 3E and Figure S3A). All fusion proteins were confirmed to be properly expressed by immunoblotting (Figure 3D,F and Figure S3C,D). These results suggested that heptad repeats were required for cell death induced by the Pi36 CC domain. In addition, a co-IP assay was also conducted to determine whether the double mutation L61E/L64E influences the self-association of the Pi36 CC domain. The self-association of the wild-type Pi36 CC domain was confirmed, whereas the CC domain with the double mutation L61E/L64E was unable to self-associate (Figure 3G). The double mutation L61E/L64E compromised both the Pi36 CC domain-mediated cell death and self-association. Overall, these results reveal that the CC domain of Pi36 can self-associate in planta, and this self-association is necessary for its cell death activity.

### 2.3. Pi36 Cell Death-Inducing Activity Is Tightly Regulated by the NB-ARC Domain

Sequence alignment with CC-NLR proteins showed that the motifs, including the P-loop/Walker A, RNBS-A, Walker B, RNBS-B, GLPL, RNBS-D, and MHD motif, were found and highly conserved in the Pi36 NB-ARC domain (Figures S1 and S5). To better understand how these motifs involve in Pi36 activation and autoinhibition, a series of mutations within these motifs were designed (Figure S5).

**Figure 4:**
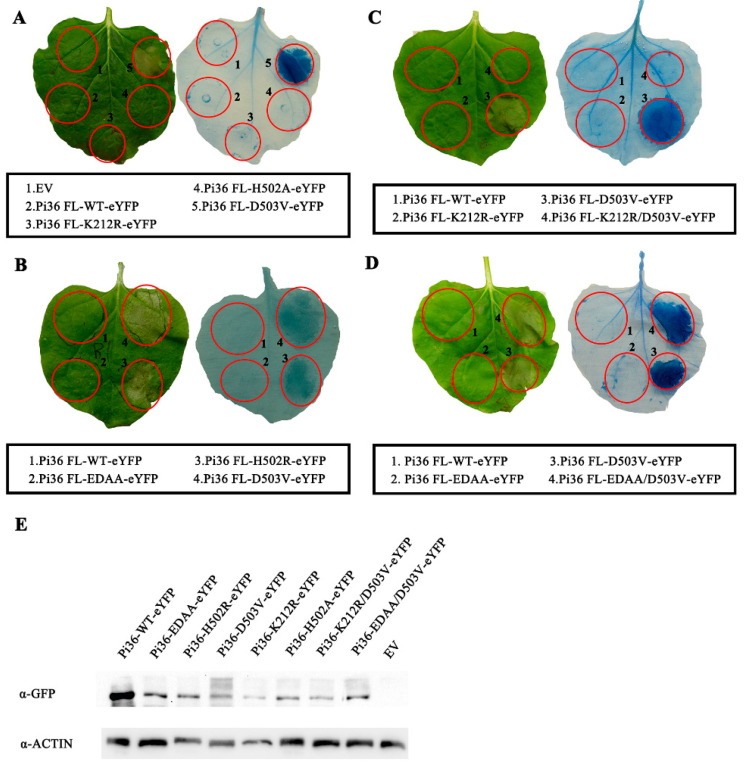
Cell death-inducing activity of Pi36 variants in *Nicotiana benthamiana.* (**A**–**D**) The cell death phenotypes of Pi36 variants with mutations in EDVID, P-loop, and MHD motifs. All Pi36 variants were C-terminally tagged with eYFP and transiently expressed in *N. benthamiana*. The mutation D503V in MHD motif autoactivated Pi36, while the mutation K212R in P-loop motif compromised this cell death activity. Pictures were taken at 7 dpi, followed by trypan blue staining. This cell death assay was repeated at least three times with similar results. (**E**) Protein expression levels of Pi36 variants were analyzed by Western blot. Total proteins extracted from *N*. *benthamiana* leaves at 20 hpi were subjected to immunoblot analysis with anti-GFP and anti-Actin antibodies.

The mutations in the P-Loop and MHD motif usually result in loss of function and constitutive activation phenotypes in plant NLRs, respectively [6,7,32,44]. A P-loop mutant, Pi36 FL-K212R, and three MHD mutants, Pi36 FL-H502A, Pi36FL-H502R, and Pi36 FL-D503V, were generated and overexpressed in *N. benthamiana* (Figure S5A,F). The Pi36 variant with the mutations H502R and D503V in the MHD motif could induce massive cell death activity in the absence of the corresponding effector, whereas another MHD mutant, P-loop variant, and Pi36-WT did not trigger any cell death phenotype, demonstrating an important role of the MHD motif in Pi36 autoinhibition (Figure 4A,B). To test whether mutation in the P-loop compromises the cell death activity of Pi36 as per previous reports, the mutation K212R in the P-loop motif was also introduced into the autoactive Pi36-D503V variant. As expected, the Pi36-K212R-D503V variant was not able to induce cell death, showing the importance of an intact P-loop or nucleotide binding in Pi36-mediated cell death (Figure 4C).

Mutations in or next to the RNBS-A, WalkerB, RNBS-B, GLPL, and RNBS-D motifs of the NB-ARC domain have been reported to compromise the cell death activity or elicit an autoactive immune phenotype without the corresponding effector in various plant NLRs (Figure S5) [12,23,40,57,58]. Related mutations were designed and also incorporated in Pi36-D503V and in Pi36-WT (Figure S5). The transient expression results in *N. benthamiana* revealed that only one mutation, D440G next to the RNBS-D motif, could induce effector-independent cell death like the mutation D503V (Figure S10A). However, the other mutations in these motifs did not exhibit such effects (Figures S6–S10). D289A and D290A in the Walker B motif, I313K next to the RNBS-B motif, and G372R and S382N in the GLPL motif blocked the cell death activity of Pi36-D503V (Figures S6, S8–S10 and Table 1). Other mutations in these motifs did not affect the states of Pi36 and Pi36-D503V. Additionally, similar to the result observed in the Pi36-D503V variant, the mutation K212R in the P-loop compromised the cell death induced by Pi36-D440G (Figure S10D,E), further demonstrating the important role of the functional P-loop motif in cell death elicitation.

Except the RNBS-A motif, structure-guided mutagenesis of these motifs in the NB-ARC domain yielded several loss-of-function and gain-of-function mutants (Table 1). The mutations D503V and D440G autoactivated Pi36 without the correspondent effector, and several mutations in the motifs of the NB-ARC domain abolished the Pi36-D503V-mediated autoactivity.

### 2.4. Pi36 Self-Association Is Constitutive and P-Loop-Dependent

In this study, self-association is essential for the cell death induced by the Pi36 CC domain (Figure 2 and Figure 3), but it remains to be determined whether the full-length Pi36 protein also self-associates and whether this self-association is essential for Pi36-mediated cell death.

**Figure 5:**
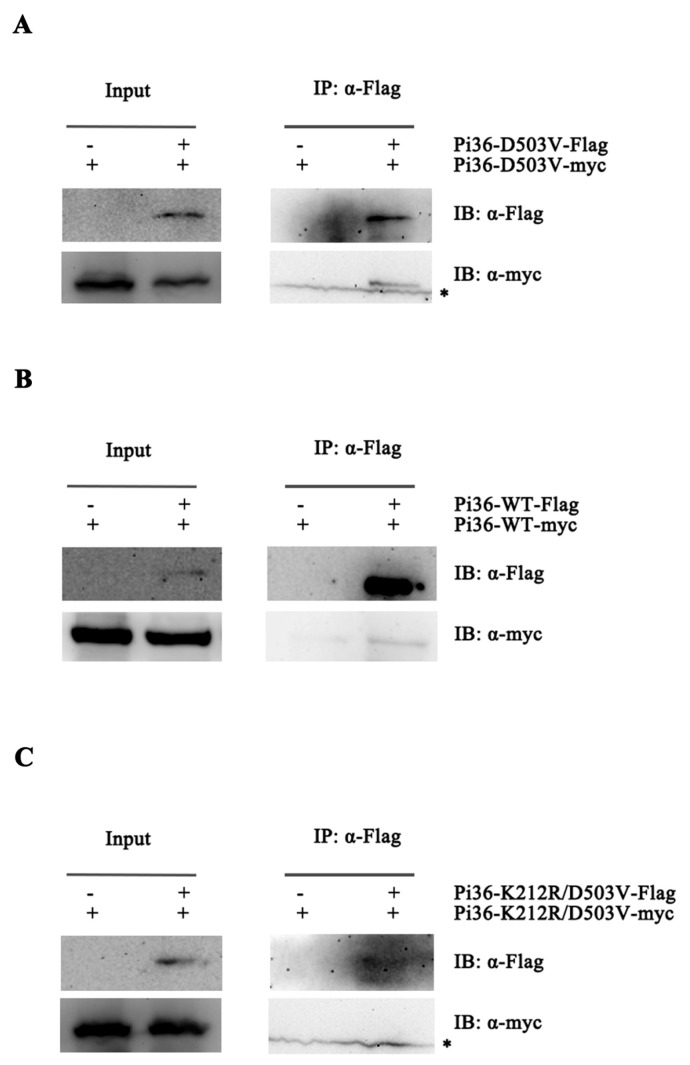
Pi36 self-associates at pre-activation and post-activation. (**A**) Wild-type Pi36 self-associated in absence of correspondent effector. (**B**) Autoactive Pi36-D503V formed self-association. (**C**) Functional P-loop motif was required for self-association of autoactive Pi36-D503V. The indicated protein combinations were transiently expressed in *N. benthamiana*. Total protein extracts from *N*. *benthamiana* leaves at 20 hpi were immunoprecipitated with anti-Flag beads, followed by immunoblotting with anti-Flag and anti-Myc antibody. The asterisk indicates non-specific bands.

The Pi36-D503V variant that elicits cell death without a correspondent effector is assumed to be in the active state. Meanwhile, Pi36-WT is assumed to be in a resting state. To elucidate the role of self-association in Pi36 activation, co-IP assays were performed to detect whether inactive Pi36-WT and autoactive Pi36-D503V form oligomers. Flag- and Myc-tagged Pi36-WT and Pi36-D503V were co-expressed in *N. benthamiana*, and the sample proteins were extracted for co-IP. Self-associations were detected both in Pi36-WT and Pi36-D503V, indicating that Pi36 can form self-associations in both the pre-activation and post-activation states (Figure 5A,B). Our early result showed that the cell death activity of Pi36-503V is P-loop-dependent (Figure 4B), and then we tested whether the self-association of Pi36-D503V is also dependent on an intact P-loop motif. As shown in Figure 5C, the self-association of Pi36-K212R-D503V was not detected, indicating that an ATP-binding or functional P-loop is not only required for the autoactivity of Pi36-D503V but also for its self-association.

### 2.5. Intermolecular and Intramolecular Interactions in Pi36

In this study, self-association has been tested to be crucial for the autoactivity conferred by Pi36-D503V and its CC domain (Figure 2, Figure 3 and Figure 5B,C), while the NB-ARC domain and NB subdomain repress the cell death activity of the CC domain (Figure 1C), raising the question of how other domains regulate CC domain-mediated autoactivity and self-association in Pi36.

**Figure 6:**
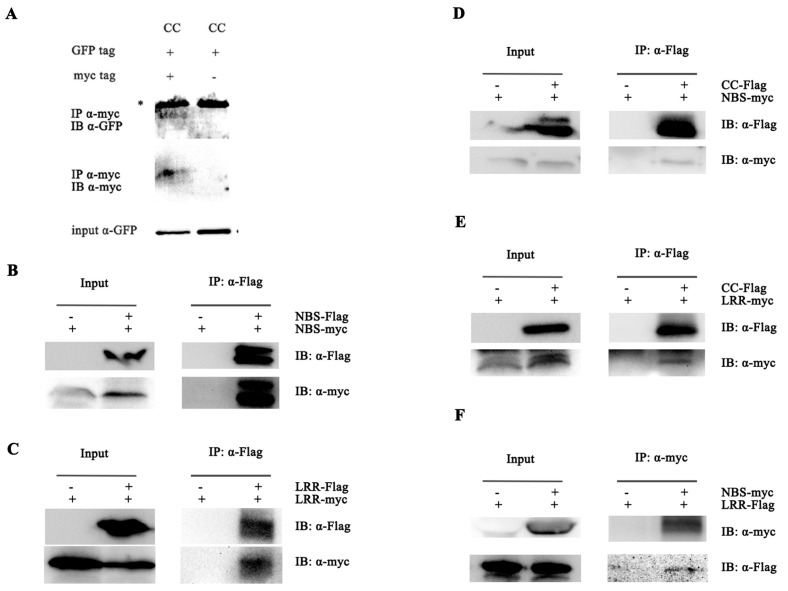
Intermolecular and intramolecular interactions of Pi36 multiple domains. Co-IP experiments were conducted to detect the interactions. Pi36 CC (**A**), LRR (**B**), and NB-ARC domain (**C**) could self-associate. CC domain interacted with NB-ARC (**D**) and LRR domain (**E**), NB-ARC domain interacted with LRR domain (**F**). Individual domain fused with different tags was transiently co-expressed in *N. benthamiana* for 20 h in indicated combinations. Proteins extracted from leaves were subjected to immunoprecipitation using anti-Myc or anti-Flag beads, followed by Western blot analysis using anti-GFP, anti-Myc, or anti-Flag antibodies. The asterisk indicates non-specific bands.

To investigate intermolecular interactions in Pi36, we tested the self-associations of its isolated domains. The CC, NB-ARC, and LRR domains of Pi36 fused to C-terminal Flag and Myc tags were overexpressed in *N. benthamiana* for subsequent co-IP assays. The assays demonstrated that the CC domain self-associated (Figure 6A), as did the NB-ARC domain (Figure 6B). The LRR-Flag was co-immunoprecipitated with LRR-Myc (Figure 6C). In the NB-ARC domain, both the NB and ARC subdomains self-associated (Figure S11A,B). Overall, our results indicate that all domains and subdomains could self-associate, suggesting that more than one domain may contribute to the self-association of Pi36.

We also conducted co-IP assays to investigate the potential interactions among these domains. As shown in Figure 6D,E, the Pi36 CC-Flag was found to interact with NB-ARC-Myc and LRR-Myc, respectively. The NB-ARC-Myc interacted with the LRR-Flag (Figure 6F). Furthermore, the Pi36 CC-Flag exhibited interactions with the NB-Myc and ARC-Myc subdomains, respectively (Figure S11A,C), while the LRR-Flag also co-immunoprecipitated with ARC-Myc as well (Figure S11D). These findings indicate that multiple domain intramolecular interactions existed in Pi36, possibly maintaining Pi36 in an inactive state. The NB-ARC domain may suppress the CC domain-mediated cell death through intramolecular interaction.

### 2.6. Autoactivity of Pi36-D503V Is Dependent on the LRR Domain

Early results have shown that several mutations in the NB-ARC domain resulted in the gain of function in Pi36-WT and the loss of function in Pi36-D503V (Table 1). In addition, some mutations were not observed to influence Pi36 and Pi36-D503V visibly. To test whether these mutations can alter the inactive state of Pi36 CC-NB-ARC, we incorporated them into the CC-NB-ARC variant and evaluated their impact on cell death.

**Figure 7:**
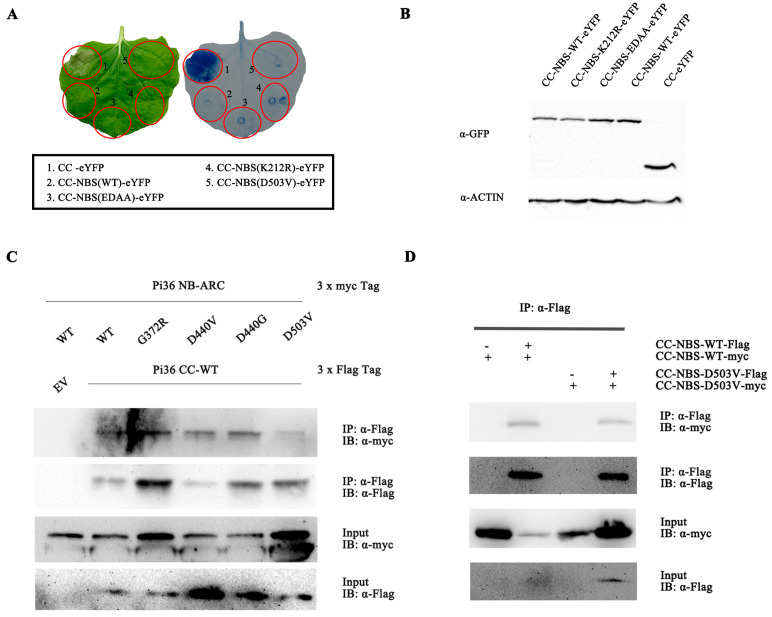
Analysis of cell death elicitation of Pi36 CC-NB-ARC variants in *N*. *benthamiana*. (**A**) The autoactive D503V could not autoactivate CC-NB-ARC(CC-NBS). (**B**) Protein expression levels of CC-NB-ARC variants. The variants were transiently expressed in *N. benthamiana*, and proteins were extracted from leaves at 20 hpi. Anti-GFP and anti-Actin antibodies were used to detect the expression of the fused proteins. Representative leaves were photographed at 7 dpi (**C**). (**D**) Self-association ability of CC-NB-ARC variants. Indicated combination of CC-NB-ARC variants was transiently co-expressed in *N*. *benthamiana* to assess the self-association by co-IP. Total proteins extracted from leaves at 20 h post-agroinfiltration and subjected to immunoprecipitation using anti-Flag beads. Detections were performed by immunoblotting with anti-Flag and anti-Myc antibodies.

Since the D503V mutation in the MHD motif mimics ATP binding, triggers conformational changes in the NB-ARC domain of Pi36, and releases the CC domain for cell death activity (Figure S4A), initially, we firstly incorporated the D503V mutation into the Pi36 CC-NB-ARC fragment to monitor cell death activity. The transient expression analysis showed that the C-terminal eYFP tag-fused Pi36 CC-NB-ARC(D503V) did not elicit cell death in *N. benthamiana* (Figure 7A). Subsequently, we introduced other mutations in motifs into the CC-NB-ARC fragment. Similar to D503V, the mutation D440G, another autoactivating mutation in Pi36 (Figure S10A), did not change the state of the CC-NB-ARC (Figure S12A). Moreover, other CC-NB-ARC variants also did not elicit cell death (Figure S12A–C). All these mutated variants exhibited a similar protein expression level as the wild-type CC-NB-ARC, indicating that these mutations may not affect the protein stability (Figure 7B and Figures S12D). Our results imply that the autoactivation of Pi36 induced by the mutations D503V and D440G requires the involvement of the LRR domain.

Co-IP assays detected the interactions between the Pi36 CC domain and NB-ARC domain carrying the mutations G372R, D440V, D440G, or D503V, indicating that these mutations did not disrupt the interaction between the CC and NB-ARC domain (Figure 7C). Overall, these selected mutations were insufficient to disrupt the suppression of the Pi36 CC domain imposed by the NB-ARC domain. Given that self-association is crucial for Pi36-mediated cell death, we performed a co-IP assay to detect whether the deletion of the LRR affects the self-association of CC-NB-ARC-D503V. The results showed that both the Pi36 CC-NB-ARC and CC-NB-ARC-D503V variants exhibited detectable self-association with similar strengths (Figure 7D), suggesting that LRR domain deletion does not inhibit autoactivation by compromising self-association.

### 2.7. Self-Association of Pi36 Is Enhanced upon Activation

**Figure 8:**
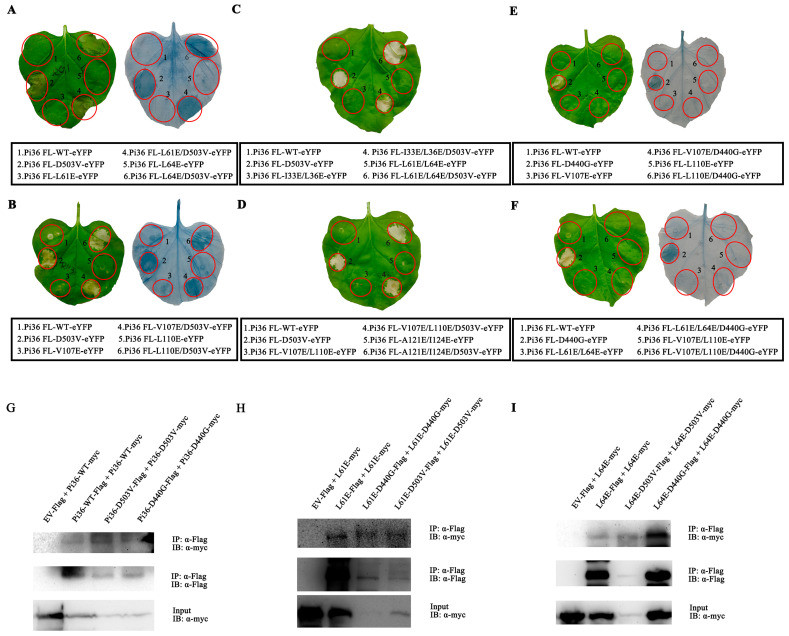
Mutations located in the CC domain exhibited opposite effects on autoactive Pi36-D440G and Pi36-D503V. (**A**–**D**) Selected mutations in CC domain could not block cell death induced by Pi36-D503V. (**E**,**F**) Selected mutations in CC domain compromised cell death induced by Pi36-D440G. All Pi36 variants were transiently expressed in *N*. *benthamiana* to assess the ability of cell death elicitation. The cell death assays were repeated at least 3 times, and representative pictures were taken at 7 dpi. (**G**–**I**) Self-association of Pi36 variants. Following the indicated combinations, Pi36 variants were transiently co-expressed in *N*. *benthamiana*. Proteins collected from infiltrated leaves at 20 hpi were immunoprecipitated with anti-Flag beads, followed by Western blot using anti-Flag and anti-Myc antibodies.

In this study, hydrophobic residues were required for the self-association and cell death mediated by the Pi36 CC domain (Figure 3 and Figure S3). However, it remains unclear whether these residues are also required for the cell death activity and self-association of Pi36. Several mutations of hydrophobic residues in the CC domain, including L61E, L64E, V107E, and L110E, were introduced into Pi36-D503V to assess their effects on cell death activities. Transient expression in *N. benthamiana* revealed that the mutations L61E, L64E, V107E, and L110E did not diminish the cell death induced by Pi36-D503V (Figure 8A,B), contrasting with the results observed in Pit and RB with MHD motif mutations [55,56]. Additional mutations in the CC domain, including the double mutations I33E/L36E, L61E/L64E, V107E/L110E, and A121E/I124E, were further tested. Consistently, these mutations also did not compromise the cell death activity of Pi36-D503V (Figure 8C,D). In contrast, V107E, L110E, L61E/L64E, and V107E/L110E impaired the cell death induced by Pi36-D440G (Figure 8E,F).

The very N-terminal MADA motif, which corresponds to the α1-helix of the CC domain, is indispensable for the immune response triggered by ZAR1, Sr35, and NRC2/3/4 [37,47,50]. The very N-terminal sequence of Pi36 somewhat resembles that of ZAR1 but is not categorized as the MADA motif (Figure S4A) [50]. According to the structure–function analyses of ZAR1, the corresponding residues L11, I12, and G16, as well as P14 and K20 in Pi36, were mutated (Figure S4A). The transient expression assays demonstrated that most mutations of these corresponding residues could abolish the Pi36 CC domain-mediated cell death (Figure S4 and Table 2). Next, these mutations were incorporated into the autoactive Pi36-D503V and Pi36-D440G to check whether they affect their cell death activity. The transient expression assays revealed that the cell death induced by Pi36-D503V and Pi36-D440G could only be compromised by the mutation L11E, whereas other mutations did not have a visible effect on the cell death activity (Figures S13 and S14 and Table 2). These results indicate that these residues have limited effects on Pi36 autoactivity, which differ from the results observed in the CC domain.

To assess the self-association ability of the Pi36 variants, a series of co-IP assays were conducted. The results revealed that the self-association of Pi36-D503V was stronger than that of Pi36-D440G, while Pi36-D440G exhibited stronger self-association than Pi36-WT (Figure 8G). The mutations L61E and L64E suppressed the cell death induced by the CC domain but not by Pi36-D503V (Figure 3E and Figure 8A). The double mutation L61E/L64E impaired the autoactivation of Pi36-D440G (Figure 8E). The self-associations of Pi36-L61E-D503V and Pi36-L64E-D503V were also stronger than those of Pi36-L61E-D440G and Pi36-L64E-D440G, respectively. In contrast, the self-associations of Pi36-L61E and Pi36-L64E were weakest in their respective groups (Figure 8H,I). Overall, the autoactive Pi36 variants exhibited stronger self-association than the inactive Pi36 variants.

In summary, nearly all the tested mutations did not compromise the cell death induced by Pi36-D503V, while a few mutations blocked the cell death induced by Pi36-D440G. Only the mutation L11E was able to block the cell death induced by CC, Pi36-D503V, and Pi36-D440G simultaneously. It appears that the active Pi36-D503V variant exhibited more tolerance to negative mutations compared to Pi36-D440G. Additionally, the residue L11 may play a crucial role in the cell death activity of Pi36 (Table 2).

## 3. Discussion

The CC domain alone induced cell death in Pi36, whereas other domains did not exhibit this activity (Figure 1). Some mutations that compromised CC-mediated cell death also disrupted the cell death induced by two autoactive variants, Pi36-D440G and Pi36-D503V (Table 2). These findings suggest that Pi36 mediated cell death activity through the CC domain, which functions as a signal component in Pi36. Self-association is reported to be required for the N-terminal domains CC or TIR-mediated cell death [7,8,59,60]. In this study, the double mutation L61E/L64E in the heptad repeats disrupted both the Pi36 CC-mediated self-association and cell death activity (Figure 3E,G). Because the heptad repeats were reported to be essential for forming binding surfaces for α-helices and facilitating helix–helix associations [6,25,55,56], we speculated that the self-association plays an important role in Pi36 CC-mediated cell death. When the self-association strength was artificially manipulated, the cell death ability of the Pi36 CC domain was correspondingly decreased or increased, respectively (Figure 2). These results indicate that self-association is indispensable for Pi36 CC domain-mediated cell death, and the strength of self-association is correlated with the cell death level. Since Pi36 mediates cell death through the CC domain, Pi36 requires the self-association of its CC domain to induce cell death.

Self-associations were detected both in the inactive Pi36-WT and active Pi36-D503V, suggesting that the oligomerization of Pi36 may not depend on its activation state (Figure 5A,B). Furthermore, both the cell death activity and self-association mediated by Pi36-D503V were lost upon introducing the P-loop mutation K212R (Figure 4B and Figure 5C), which is consistent with previous reports in RPP1, ZAR1, Tm-2^2^, and RPM1 [25,28,37,46]. Our results suggest that Pi36-D503V-mediated cell death is also dependent on its self-association. Interestingly, the self-association strength of Pi36-D503V was found to be stronger than that of Pi36-D440G, and the self-association strength of Pi36-D440G was stronger than that of Pi36-WT (Figure 8G). Additional assays further confirmed that the autoactive Pi36 variants displayed stronger self-association compared to the inactive variants, and the mutation D503V drove a stronger self-association than D440G (Figure 8H,I). As discussed earlier, Pi36 requires the self-association of its CC domain to induce cell death (Figure 2 and Figure 3). We speculated that the stronger self-association of Pi36 during activation may support the self-association of the CC domain more effectively. This may explain why most loss-of-function mutations in the CC domain did not compromise the Pi36-D503V activity (Table 2). Mutations in the hydrophobic residues of the CC domain compromised the cell death induced by Pi36-D440G, presumably due to its insufficient oligomerization ability.

In addition to the CC domain, the NB-ARC, and LRR domains could form self-associations, and the NB and ARC subdomains could also self-associate. Similar results have been reported for other NLRs, such as Rp1-D21, Tm-2^2^, Pm21, ZAR1, and RPM1 (Figure 6 and Figure S11) [7,25,28,43,44]. Pi36 requires the self-association of its CC domain to induce cell death. The mutation D503V in the NB-ARC domain strengthened the self-association of Pi36 (Figure 8G), whereas a P-loop mutation in the same domain disrupted this self-association (Figure 5B,C), highlighting the significant role of the NB-ARC domain in self-association. However, the mutation D503V could not enhance the self-association of the CC-NB-ARC variant like it did for the full-length Pi36 (Figure 7D and Figure 8G). Given that the LRR domain also possesses self-association ability (Figure 6C), it may also help Pi36 to enhance its self-association during activation. Taken together, the enhanced self-association of Pi36 relies on all three domains during activation.

The MHD motif binds the β-phosphate of ADP, and mutations in this motif have been shown to lead to autoactivation in several plant NLRs [6,16,31,32]. The Walker-A motif/P-loop motif that interacts with ADP and the β-phosphate of ATP, and the RNBS-B motif that contracts the γ-phosphate of ATP, are both required for immune function [1,12,18,19,35,36,37,38,39]. Consistent with these findings, the mutation D503V in the MHD motif autoactivated Pi36 (Figure 4A,B), while the cell death mediated by Pi36-D503V was compromised by K212R in the P-loop motif and I313K in the RNBS-B motif (Figure 4C and Figure S8D). Additionally, the Pi36-D440G-mediated cell death was also compromised by K212R in the P-loop motif (Figure S10D). These results suggest that ATP binding or a functional P-loop is critical for Pi36-mediated cell death activity and support the notion that ADP-bound NLRs are in an inactive state, while ATP-bound NLRs are in an active state. The NB-ARC domain can hydrolyze ATP to ADP, transitioning NLRs from an active to an inactive conformation [1]. In some cases, the ATP hydrolysis ability is crucial for NLR immune function. Interestingly, mutations in the RNBS-A motif of Rht-B13b and I-2, as well as in the Walker B motif of RPS5, I-2, and SUT1, lead to autoactivation due to impaired ATP hydrolysis activity, showing that the status of nucleotide binding may be more critical than the ATP hydrolysis activity [12,23,40,41]. However, none of the designed mutations in these two motifs could autoactivate Pi36. Only the mutations D289A and D290A in the Walker B motif compromised the cell death activity of Pi36-D503V (Figures S6 and S7 and Table 1). Since no visible signs of cell death were observed in the Pi36 mutants, and biochemical analysis was not conducted in these mutants, it remains unclear how the ATP hydrolysis ability of the NB-ARC domain is involved in Pi36 activation and autoinhibition. Taken together, the NB-ARC domain acts as a molecular switch to regulate the cell death activity of Pi36, and the nucleotide-binding state is critical for the Pi36 state.

Similar to I-2, ZAR1, Sr35, and RPP1, a truncated version of Pi36 without the LRR domain did not induce cell death activity (Figure 1C) [10,12,43,46]. I-2N, the truncated form of I-2 without the LRR domain, exhibited a higher affinity for ADP than ATP [12]. This result suggests that the conformation of the Pi36 NB-ARC domain was not suitable for nucleotide exchange in the absence of the LRR domain, and a force driving conformational change for ADP-ATP exchange is required in Pi36 activation. Previous studies have proposed an allosteric mechanism involving “steric clash” with the NB domain, triggering structural remodeling of the NB domain and resulting in the exchange of ADP for ATP [13,17,19,47]. It is likely that Pi36 employs a similar mechanism within its NB domain to facilitate nucleotide exchange during activation. As described in previous reports, the WHD-LRR fragment moved away from the nucleotide-binding site during NLR activation, displacing the MHD motif within the WHD domain. This conformational change stabilizes ATP binding and exposes the oligomerization interface [17,18,37,47]. The mutations D440G and D503V in the WHD subdomain (C-terminal part of the ARC domain) autoactivated the full-length Pi36 (Figure 4A and Figure S10A), but not the CC-NB-ARC variant (Figure 7A and Figure S12A), suggesting that there is an inhibitory role in WHD, and structural reorganization of the WHD domain is required to release this inhibition during Pi36 activation. Since the CC-NB-ARC variants with the autoactive mutation D440G or D503V could not induce cell death, the LRR domain appears to be necessary for the structural reorganization of the WHD subdomain in Pi36. The LRR domain likely drives the rotation of the WHD subdomain from the nucleotide-binding site, stabilizing ATP binding and exposing the oligomerization interface. This idea effectively explains the observation that the self-association of CC-NB-ARC was not enhanced like the full-length Pi36 protein when the mutation D503V was introduced (Figure 7D). The removal of the LRR mayprevent the exposure of the oligomerization surface. In summary, Pi36 likely undergoes a series of conformational changes that involve effector perception, the exchange of ADP for ATP, and self-association enhancement, which is reminiscent of the ZAR1 and Sr35 model. These changes are mainly mediated by the NB-ARC domain for promoting ADP-ATP exchange and driving self-association. The structural remodeling in the LRR domain may help NB-ARC to release the repression of ADP-ATP exchange and expose the oligomerization surface. Although an SNP in the LRR domain is associated with the resistant phenotype of Pi36, whether the LRR domain recognizes the effector and guides “steric clash” is unclear [48].

Cell death and disease resistance elicited by activated plant NLRs must be tightly controlled in the absence of pathogens to avoid deleterious effects [61]. NLRs are often maintained in an “off” state through intramolecular interactions, which are illustrated by the inactive ZAR1-RKS1 complex structures, and structure-guided analysis of RPM1, Tm-2^2^, RPS5, and Rx [8,13,14,24,25,28,43]. For Pi36, the CC domain interacted with the NB-ARC and LRR domains, and the NB-ARC and LRR domains also associated with each other (Figure 6D–F). Moreover, interactions between the CC and NB domains, the CC and ARC domains, and the LRR and ARC domains have also been observed in Pi36 (Figure S11A,C,D). The complex intramolecular interactions in Pi36 seem to fit well with previous reports, which describe how to maintain the NLRs in an inactive state. The interactions between the CC domain with multiple other domains maintain the CC domain in an inactive state [13,25,28,43]. In Pi36, the CC domain induced cell death, while CC-NB induced weak cell death; however, CC-NB-ARC could not induce cell death (Figure 1). The CC domain of Pi36 interacted with the NB-ARC domain, including its subdomains NB and ARC (Figure 6C and Figure S11A,C). Thus, it is reasonable to infer that the interaction between the CC and NB-ARC domains keeps the CC domain of Pi36 inactive. The WHD-ADP interaction is reported to have an inhibitory role in NLR regulation [13,16,31,33]. The mutation D503V in the MHD motif and D440G next to the RNBS-D motif, both located in the WHD domain, autoactivated Pi36 (Figure 4A and Figure S10A). The LRR domain is required for Pi36-D440G- and Pi36-D503V-mediated cell death (Figure 7A and Figure S12A). These results suggest that the WHD-ADP interaction also has an inhibitory role in Pi36 activation, and the position of the WHD subdomain is critical for stabilizing the inactive Pi36.

Here, we propose a model for Pi36 autoactivation and activation. In the inactive state, the NB-ARC (comprising the NB-HD1-WHD module) domain interacts with the CC domain to repress its autoactivation. Upon ligand perception, structural remodeling may occur in the NB domain to facilitate nucleotide exchange. Subsequently, ATP binding causes conformational changes in multiple domains, changing their relative positions. The LRR domain is hypothesized to displace the WHD from the nucleotide-binding pocket, thereby stabilizing ATP binding and exposing the oligomerization surface. The ATP-bound NB-ARC domain releases the repression on the CC domain and promotes the stronger self-association of Pi36. This enhanced self-association of Pi36 brings derepressed CC domains into close proximity, ultimately leading to cell death. During Pi36 activation, the nucleotide exchange, self-association, and potential conformational changes show similarities to the ZAR1 and Sr35 model [13,19,47]. The ATP binding is shown to be crucial for the self-association and cell death activity of Pi36, ZAR1, and Sr35. In the cases of ZAR1 and Sr35, their oligomerizations also support their CC domains in forming funnel-shaped structures for channel activity. Similarly, the self-association is enhanced to support the CC domain during Pi36 activation. However, it remains unclear whether Pi36 oligomerizes into pentamers like ZAR1 and Sr35, and whether the CC domain of Pi36 forms funnel-shaped structures as observed in ZAR1 and Sr35. Our speculation on the potential conformational changes in Pi36 is based on structure-guided analysis and the ZAR1 and Sr35 model, though differences between Pi36 and these models are uncertain. In the ZAR1 and Sr35 models, plasma membrane (PM) localization and N-terminus exposure are required for their function or their CC domain function; however, these requirements for Pi36 remain unclear and need to be elucidated.

Due to the observed autoactivation in the Pi36 variants and CC domain, it might be feasible to drive them under an inducible promoter or artificial promoter in rice to achieve the broad-spectrum resistance to rice blast fungal. The Pi36 variants or CC variants that confer a weak cell death activity may elevate resistance with fewer fitness costs. In addition, transferring Pi36 between plant species may be another pathway to expand disease-resistance specificities. Recent advances in extending or changing effector recognition through NLR engineering present new opportunities for the creation of disease-resistant genes. These successful implementations benefit from the precise structural knowledge of the interaction between the NLR proteins and effector. However, the relationship between Pi36 and its correspondent effector remains unclear and needs to be elucidated.

## 4. Materials and Methods

### 4.1. Plasmid Construction

Three pBI211, pCAMBIA1300, and pCAMBIA2300 were ligated with a 35S promoter- MCS- NOS terminal cassette. Tags eYFP, 3×Flag, and 3×Myc were cloned into C-terminal MCS of pBI211, pCAMBIA1300, and pCAMBIA2300, respectively. The modified vector pBI211 with eYFP tag was used for assessing the cell death activity, while the modified pCAMBIA1300 and pCAMBIA2300 were used for co-IP assays. The expression cassettes and Pi36-related fragments were ligated into these binary vectors using a ClonExpress II One Step Cloning Kit (C122-01, Vazyme, Nanjing, China). The Phanta Max Super-Fidelity DNA Polymerase (P505-d1, Vazyme, China) was used to amplify all fragments for plasmid construction, and all constructs were confirmed by DNA sequencing (Sangon, Shanghai, China).

### 4.2. Site-Directed Mutagenesis

Target fragments were cloned into pEASY^®^-Blunt Simple Cloning Vector (CB111-01, Transgen, Beijing, China), and high-purity plasmids were served as PCR templates for site-directed mutagenesis. Complementary primers carrying desired mutations were designed to amplify the plasmid templates through 20 cycles in a 50 μL reaction using the Phanta Max Super-Fidelity DNA Polymerase (P505-d1, Vazyme, China). An amount of 1 μL PCR products was treated with DpnI at 37 °C in a 20 μL reaction for 1 h to digest the methylated plasmid DNA templates in vitro. Next, 2 μL of DpnI-treated products was incubated with the homologous recombination enzyme (C112-01, Vazyme, China) at 37 ℃ in a 20 μL reaction for 30 min to achieve the cyclization of linearized DNA. To enhance the mutation efficiency further, 10 μL of recombination products was directly transformed into the DMT *E. coli* cell to digest the potential template plasmid in vivo. All constructs were sequenced to verify the mutated fragments.

### 4.3. Agrobacteria-Mediated Transient Expression

Agrobacterium-mediated transient expression (agroinfiltration) was performed as described previously with minor modifications [50]. The tag fusion constructs were transformed into *Agrobacterium tumefaciens* strain GV3101. The *Agrobacterium tumefaciens* bacteria were cultured in the dark at 28 °C for 2 d on LB agar plates with appreciate antibiotics. A positive bacterial clone was transferred into 3 mL LB liquid medium with antibiotics, and then cultured in the dark at 28 °C/200 rpm for 1~2 d. The bacteria were collected at 5000× *g* rpm by centrifugation and resuspended in MES buffer (10 mM MES pH5.6, 10 mM MgCl_2,_ and 200 μM acetosyringone). The final concentration of each strain was adjusted to an OD_600_ 0.5 for the cell death activity test and an OD_600_ 1.0 for the co-IP assays. The resuspended solutions were incubated in the dark at room temperature for another 3 h before infiltration. About 4–5-week-old *N. benthamiana* leaves were selected to be infiltrated. For the cell death activity test, at least 10 individual leaves from different leaves were infiltrated for each construct, and the experiment was repeated at least three times using a different positive bacterial clone.

### 4.4. Protein Analysis

For the protein expression analysis for the cell death activity test, the leaf samples were collected as described previously [50]. In brief, 0.1 g of infiltrated leaves was collected at 20 h post-infiltration (hpi). The samples were ground into fine powder using liquid nitrogen, and total protein was homogenized in 200 μL protein extraction buffer (10% Glycerol, 25 mM Tris-HCl pH 7.5, 1 mM EDTA, 150 mM NaCl, 10 mM DTT, 0.5% Nodinet-40, 1 × Protease inhibitor cocktail) [28]. And then the lysates were incubated on ice for 30 min, followed by centrifugation at 12,000× *g* for 30 min at 4 °C. The obtained supernatants were loaded for SDS-PAGE, and the eYFP-, Flag-, and Myc-tagged proteins were detected by immunoblotting using anti-GFP antibody (MA5-15256, Invitrogen, Waltham, MA, USA, 1:2000 (*v*/*v*)), anti-Flag antibody (M20008, Abmart, Shanghai, China, 1:5000 (*v*/*v*)), and anti-Myc antibody (M20002, Abmart, China, 1:5000 (*v*/*v*)), respectively, and followed hybridization with the second antibody anti-mouse IgG conjugated with HRP (D110087, Sangon, China 1:5000 (*v*/*v*)). The HRP signal was detected by ECL substrate kit (P0018AS, Beyotime, China) in ChemiDoc system (Bio-rad, New York, NY, USA).

At least 1g leaf tissues expressing two constructs with interest genes was collected at 20 hpi for the co-IP assays. Samples were ground in liquid nitrogen and homogenized in 2 mL of protein extraction buffer. The lysates were also incubated on ice for 30 min, followed by centrifugation at 12,000× *g* at 4 °C until the supernatants were clear. An amount of 50 μL of supernatants was collected as input samples. The residual supernatants were mixed with 20 μL of Anti-Flag, -GFP, or -Myc Magnetic Beads (P2115/P2132/P2118, Beyotime, Shanghai, China), and incubated with end-over-end turning at 4 °C for 12 h. After being washed 4~5 times using prechilled protein extraction buffer, beads were inoculated in 50~100 μL of elution buffer (0.1M Glycine-HCl, pH3.0) for 20 min at 4 °C, followed by mixing with 5~10 μL of neutralize buffer (0.5M Tris-HCl, pH7.4, 1.5M NaCl). Finally, the inputs and immunopurified substrates were detected by immunoblot as described above.

## 5. Conclusions

In the present work, we reported that Pi36 activation involves both the nucleotide exchange of ADP for ATP and self-association. Furthermore, this activation process is predicted to be accompanied by sequential conformational changes. The NB-ARC acts as a molecular switch, regulating the activation of the CC domain via nucleotide exchange and enhanced self-association with the help of the LRR domain. Our findings shed some light on the role of Pi36 in cell death regulation. However, further investigations are needed to elucidate the link between Pi36 activation and disease-resistance induction.

## Figures and Tables

**Table 1:** Mutations in the NB-ARC domain yielded gain-of-function and loss-of-function mutants.

Domain	Motif	Mutations Autoactivate Pi36	Mutations Inactivate Pi36-D503V
NB	P-loop		K212R
	RNBS-A		
	Walker B		D289A, D290A
	RNBS-B		I313K
ARC	GLPL		G372R, S382N
	RNBS-D	D440G	
	MHD	D503V	

The blank represents that no mutations in this motif autoactivated Pi36 or inactivated Pi36-D503V. The data presented in this table were extracted from Figure 4 and Figures S6–S10.

**Table 2:** Summary of cell death phenotypes conferred by Pi36 CC, Pi36-D503V, and Pi36-D440G with mutations located in the CC domain.

Introduced Mutations	Pi36 CC Domain	Pi36-D503V	Pi36-D440G
	HR	HR	—
L61E	Weak HR	HR	—
L64E		HR	—
V107E		HR	
L110E	HR	HR	
I33E + L36E		HR	—
L61E + L64E		HR	
V107E + L110E		HR	
A121E + I124E		HR	—
L11A	HR	HR	HR
L11E			
I12A	HR	HR	HR
I12E		HR	HR
G16A	HR	HR	HR
G16E		HR	HR
P13A		HR	HR
P13E		HR	HR
K20A	HR	HR	HR
K20E		HR	HR

“—” indicates that mutations were not tested. “HR” indicates that the autoactive Pi36 CC, Pi36- D503V, or Pi36-D440G with mutations at CC domain could still induce cell death. Blank represents those mutations compromised the cell death activity. The data presented in this table were extracted from Figure 8, Figures S13 and S14.

## Data Availability

Data are contained within the article and Supplementary Materials.

## Supplementary Materials

The following supporting information can be downloaded at: https://www.mdpi.com/article/10.3390/ijms25137301/s1.
