# Supplementary material for: Activation and Autoinhibition Mechanisms of NLR Immune Receptor Pi36 in Rice
Source: Int J Mol Sci. 2024 Jul 2;25(13):7301. doi: 10.3390/ijms25137301 (PMC11242311; doi:10.3390/ijms25137301)
Supplement: Supplementary file 1 [file ijms-25-07301-s001.zip › ijms-3071099-supplementary.pdf]

Supplementary material

Figure S1. Multiple sequence alignment of Pi36 and MLA family proteins.
Figure S2. Transiently expressed of Pi36 truncation proteins in *N. benthamiana*.
Figure S3. Impact analysis of more hydrophobic residues in Pi36 CC mediated cell death.
Figure S4. Very N-terminus is required for Pi36 CC mediated cell death.
Figure S5. Potential autoactivating or inactivating mutations in Pi36.
Figure S6. Impact of mutations in RNBS-A motif on cell death induction of Pi36.
Figure S7. Impact of mutations in Walker B motif to the cell death induction of Pi36.
Figure S8. Cell death induction analysis of Pi36 with mutations in RNBS-B motif.
Figure S9. Analysis of cell death induction of Pi36 variants with mutations GLPL motif in *Nicotiana benthamiana*.
Figure S10. Analysis of cell death induction of Pi36 variants with mutations RNBS-D motif in *Nicotiana benthamiana*.
Figure S11. Interactions among Pi36 domains.
Figure S12. Analysis of cell death elicitation of additional CC-NB-ARC variants in *N.benthamiana*.
Figure S13. Investigating roles of residue L11 in Pi36 mediated cell death.
Figure S14. Investigating roles of residue P13 in Pi36 mediated cell death.

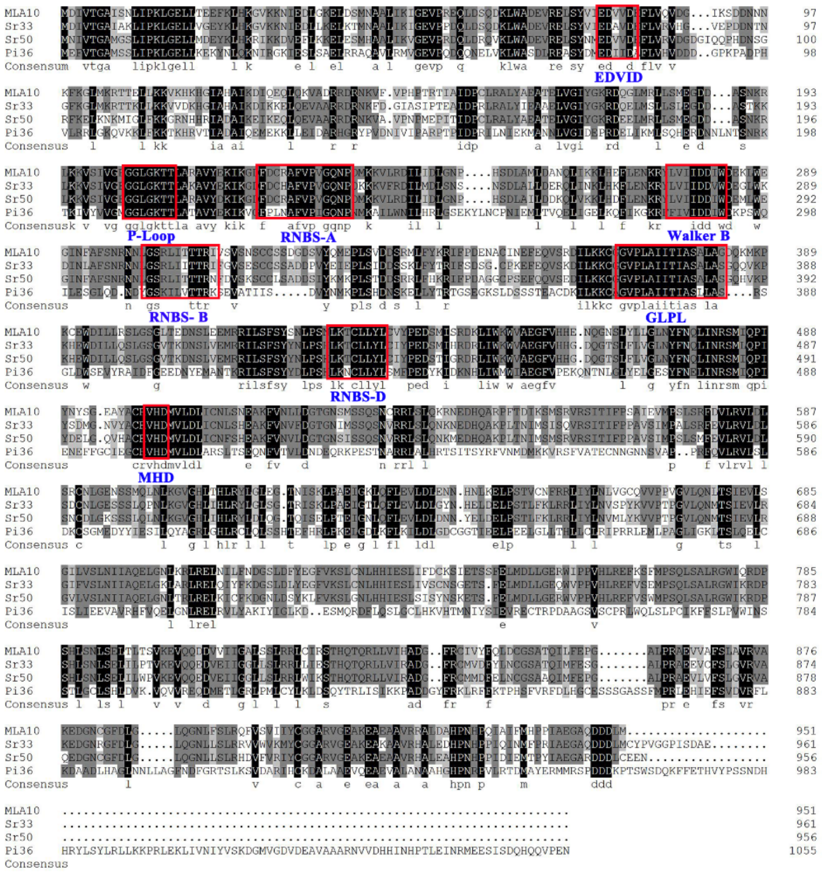

Figure S1. Multiple sequence alignment of Pi36 and MLA family proteins. Amino acid alignment of Pi36 with MLA family proteins Sr33, Sr50 and MLA10. The amino acids of conserved motifs are indicated in red box, and labeled below the sequences. The black, dark gray, light gray shaded regions represent 100%, above 75%, and above 50% similarity of the amino acids, respectively. Sequence data can be gained from the GenBank database. The accession numbers: Sr33 (AGQ17382.1), Sr50 protein (ALO61074.1), MLA10 protein (AAQ55541.1), and Pi36 (ADF29623.1)

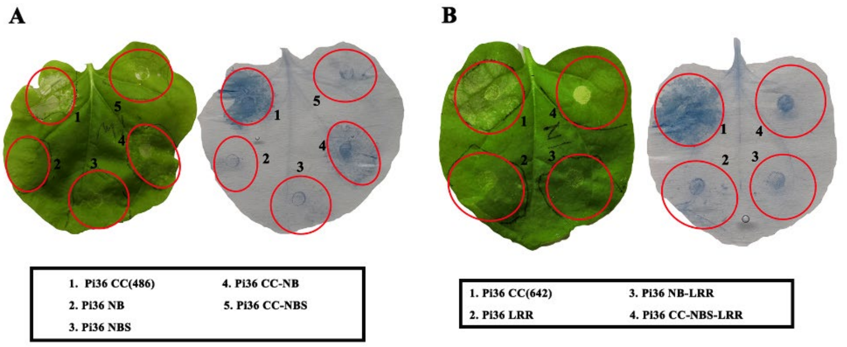

Figure S2. Transiently expressed of Pi36 truncation proteins in *N. benthamiana*. (A-B) Cell death phenotype of Pi36 fragments without any tags in *N. benthamiana*. All fragments of Pi36 were transiently expressed in *N. benthamiana*. Similar with fragments fused C-terminal eYFP, the CC domain induced strong cell death, CC-NB induced weak cell death, and other fragments or domains had no visible phenotypes. Pictures were photographed at 7 days post infiltration (dpi), followed by trypan blue staining. This experiment was repeated at least 3 times.

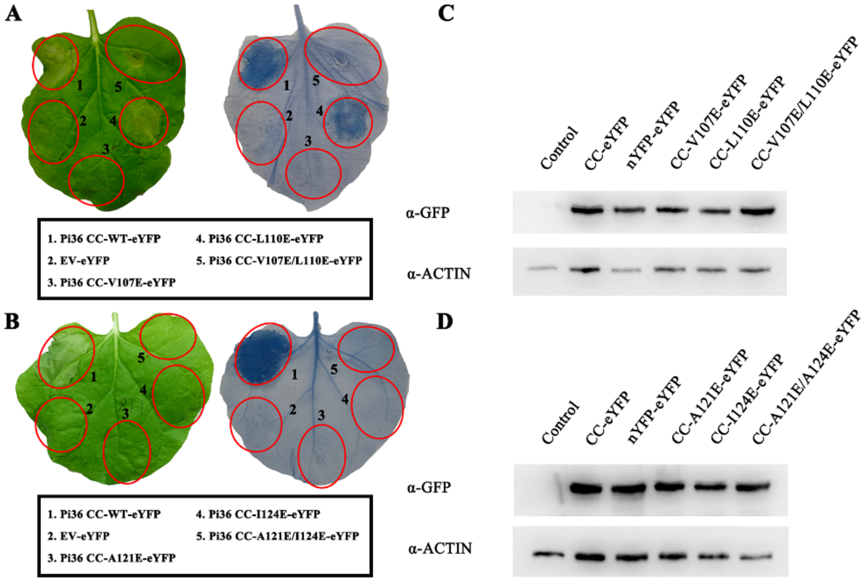

Figure S3. Impact analysis of more hydrophobic residues in Pi36 CC mediated cell death. (A-B) additional mutations of hydrophobic residues suppressed cell death induced by Pi36 CC domain. Pi36 CC variants were transiently expressed in *N.benthamiana* to monitor the cell death elicitation. This experiment was conducted at least 3 times with similar results, and the visible phenotypes were photographed at 7dpi. (C-D) Protein expression levels of Pi36 CC mutants by western blot. Total proteins extracted from agroinfiltrated *N.benthamiana* leaves at 20hpi, were subjected to immunoblot analysis using anti-GFP and anti-Actin antibodies.

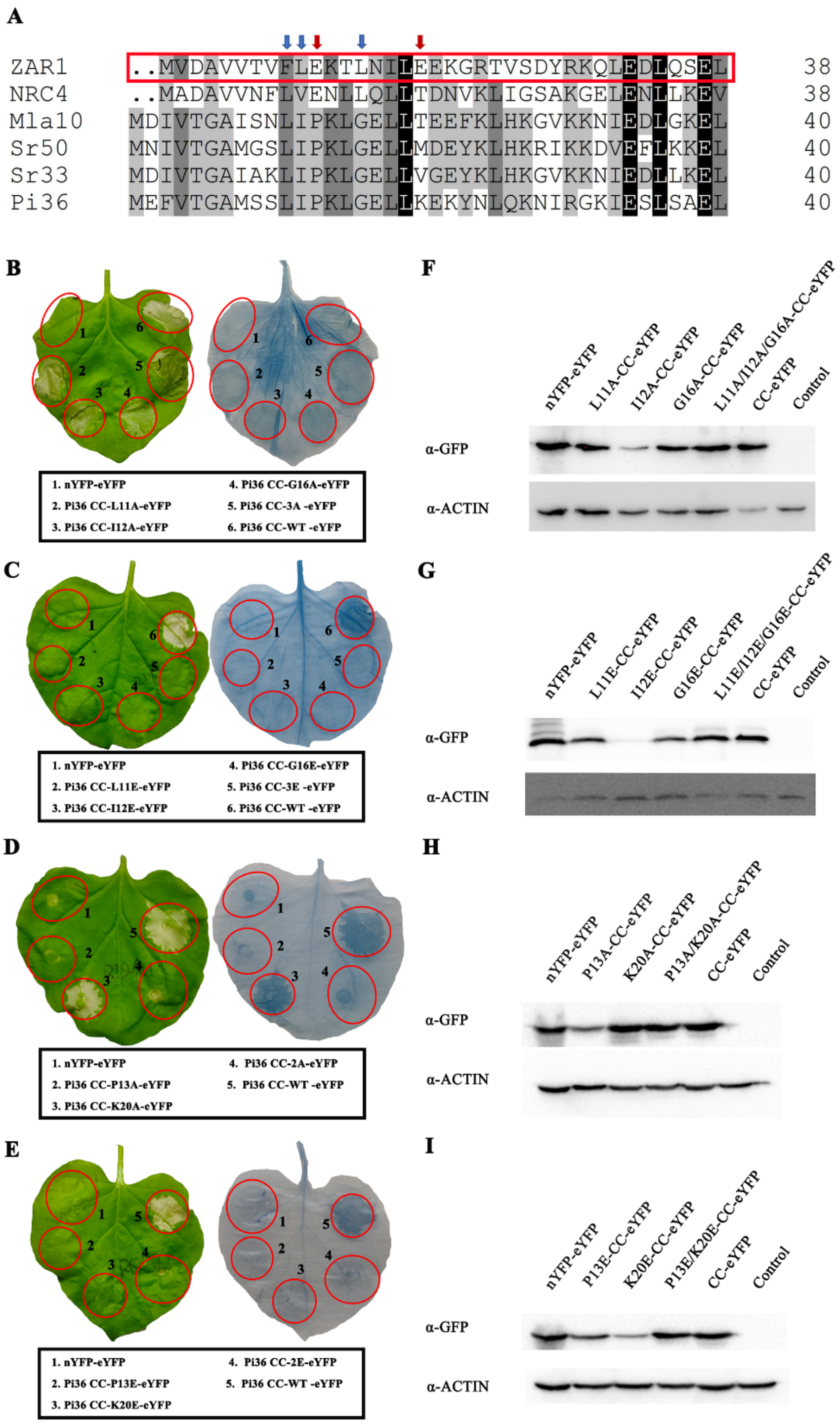

Figure S4. Very N-terminus is required for Pi36 CC mediated cell death. (A) Sequence alignment of ZAR1, NRC4, MLA10, Sr33, Sr50 and Pi36 in very N-terminal CC domain. The functional sequence in very N-terminal ZAR1 was marked by red box, and the critical hydrophilic and hydrophobic residues were pointed by red and blue arrows. (B-E) Mutations in N-terminus abolished cell death induced by Pi36 CC domain. Correspondent residue L11, I12, G16, P13 and K20 were mutated to test cell death elicitation. The CC variants were transiently expressed in *N.benthamiana*, and pictures were taken at 7dpi. This experiment was repeated at least 3 times with similar results. (F-I) Protein expression levels of Pi36 CC mutants by western

blot. Total proteins extracted from agroinfiltrated leaves at 20hpi, were subjected to immunoblot using Anti-GFP and anti-Actin antibodies.

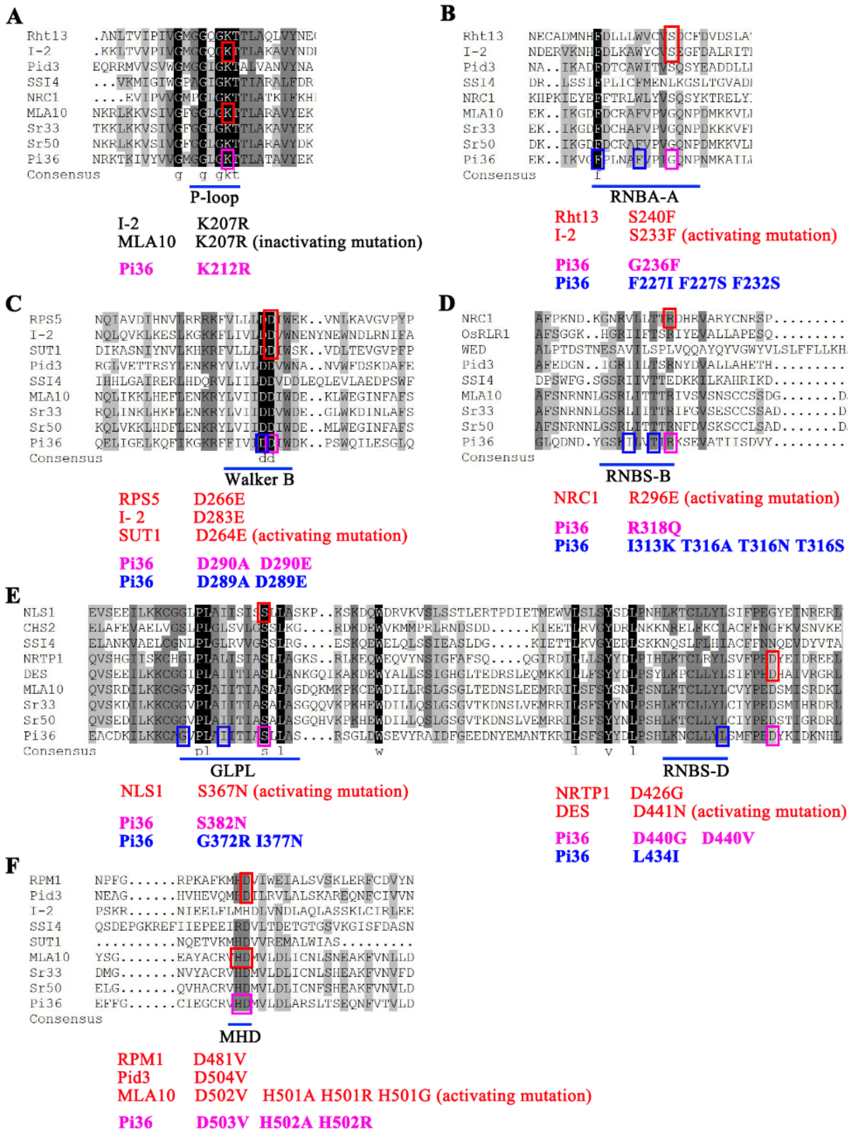

Figure S5. Potential autoactivating or inactivating mutations in Pi36. Sequence alignment of Pi36 and multiple plant NLRs in conserved motifs. The conserved motifs are underlined by blue bars and labeled below the sequences. The mutated amino acids that autoactivating or inactivating NLRs in or nest to motifs are demonstrated by red boxes, and amino acid changes were also described in red or black fonts below the aligned sequences, respectively. With reference to these reported mutations, corresponding amino acids in Pi36 were indicated by pink boxes, and mutations were described in pink fonts. Conserved amino acids in motifs were selected and boxed in blue color, and the mutations are labeled in blue fonts.

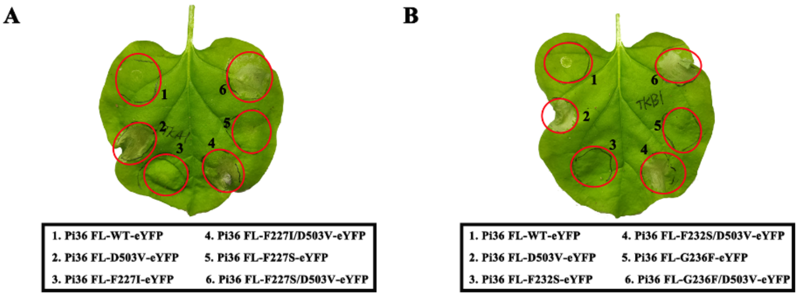

Figure S6. Impact of mutations in RNBS-A motif on cell death induction of Pi36. (A-B) Cell death phenotypes of autoactive Pi36-D503V and wild-type Pi36 with mutations in RNBS-A motif. The Pi36 mutants were transiently expressed in *N.benthamiana* to assess the cell death elicitation, and representative leaves were photographed at 7 dpi. The selected mutations F227I, F227S, F232S and G236F could not autoactivate Pi36, and also could not compromise the cell death induced by Pi36-D503V. This experiment was repeated at least 3 times with similar results.

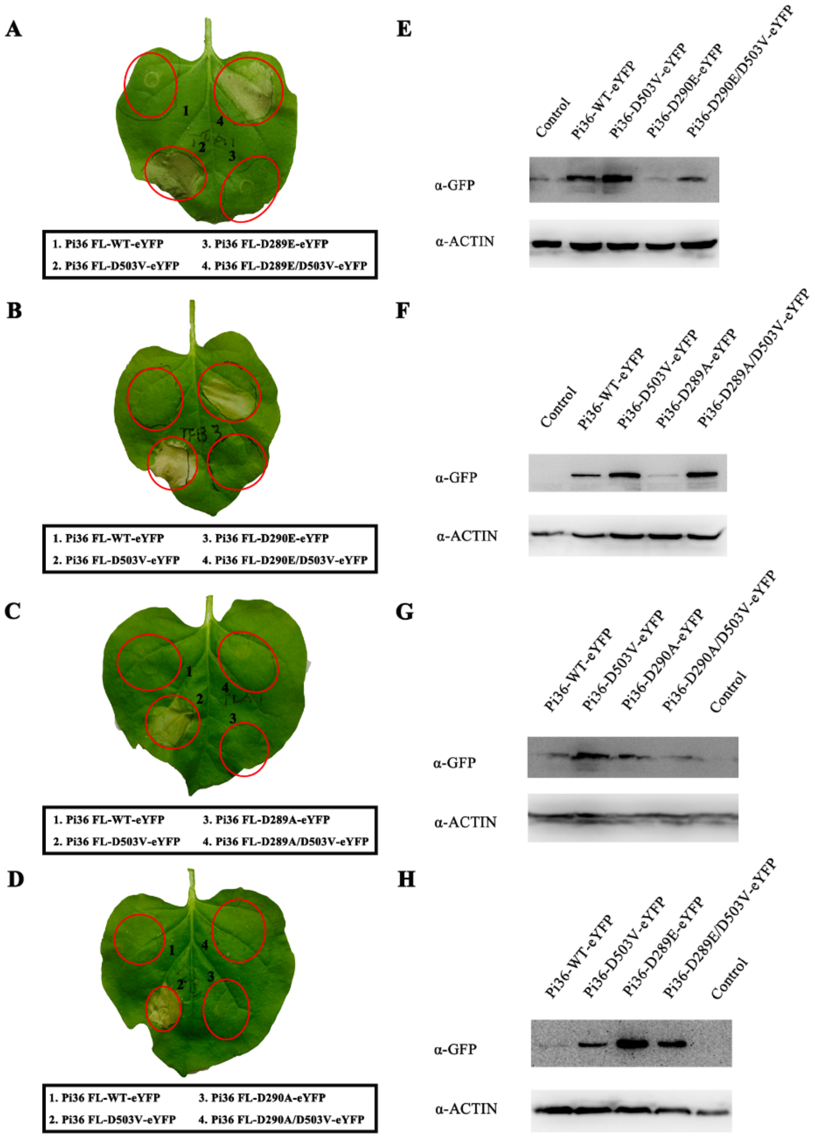

Figure S7. Impact of mutations in Walker B motif to the cell death induction of Pi36. (A-D) The cell death phenotype of Pi36 variants with mutations in Walker B motif. All the Pi36 variants were fused with a C-terminal eYFP tag and transiently expressed in *N. benthamiana*. Selected mutations D289E, D290E, D289A and D290A could autoactivated Pi36. D289A and D290A compromised the cell death induced by Pi36-D503V. This experiment was repeated at least 3 times with similar results. Representative pictures were taken at 7dpi. (E-H) Protein expression levels of Pi36 variants with mutations in Walker B motif. The Pi36 variants constructs were transiently expressed in *N.benthamiana*, and proteins were extracted from leaves at 20hpi. Anti-GFP and anti-Actin antibodies were used to detect the expression of the fused proteins.

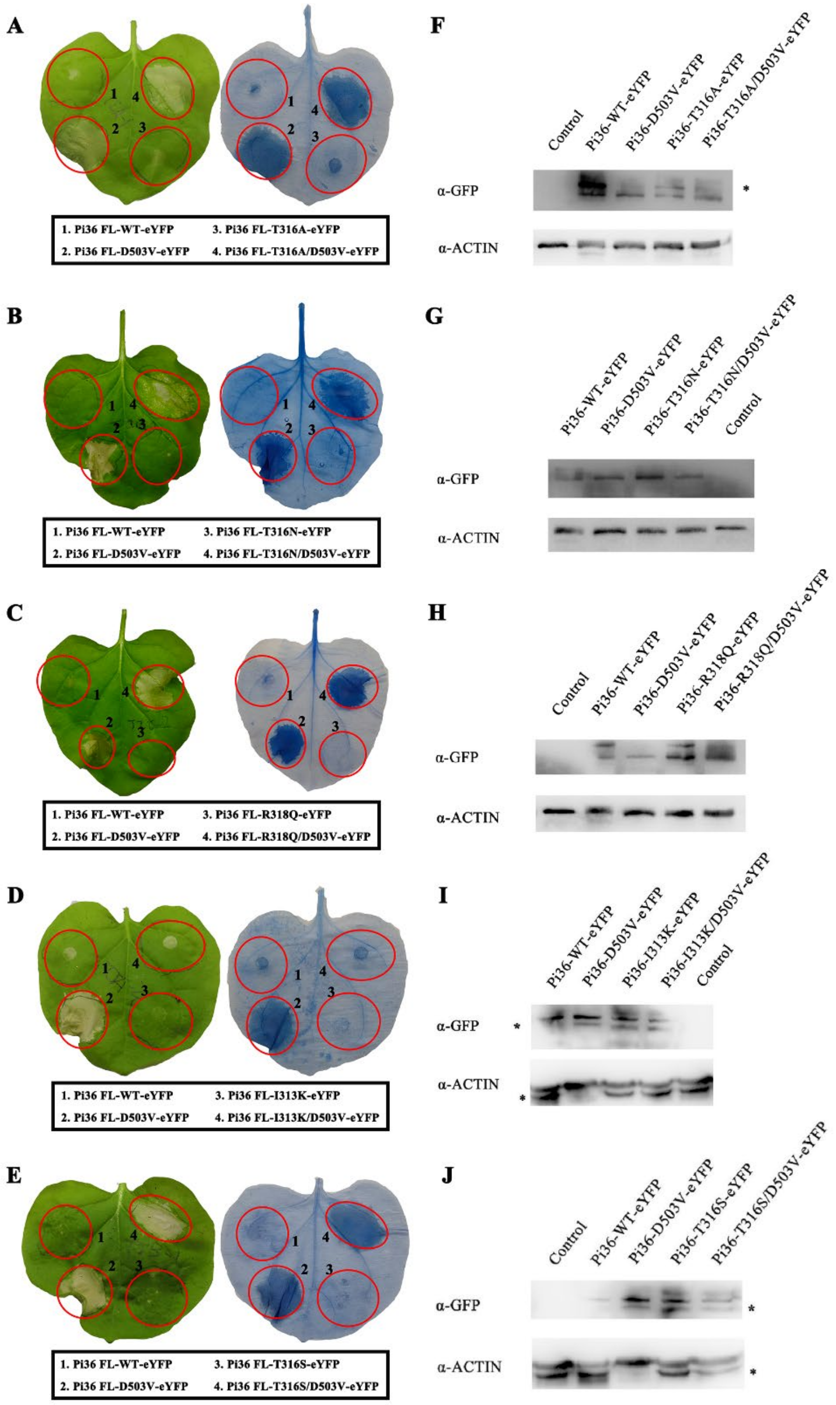

Figure S8. Cell death induction analysis of Pi36 with mutations in RNBS-B motif. (A-E) The cell death phenotype of Pi36 variants containing mutations in RNBS-B motif. C-terminal tagged Pi36 variants were transiently expressed in *N. benthamiana* by agroinfiltration. Designed mutation I313K, T316A, T316S, T316S and R318Q could not autoactivated Pi36, but cell death induced by Pi36-D503V was perturbed by mutation I313K. This experiment was repeated at least 3 times with similar results. The representative leaves were photographed at 7dpi. (F-J) Protein expression levels of Pi36 variants with mutations in RNBS-B motif by western blot. Total protein was extracted from agroinfiltrated leaves at 20hpi, and anti-GFP and anti-Actin antibodies were used to detect the expression of the fused proteins through western blot. The asterisk indicates non-specific bands.

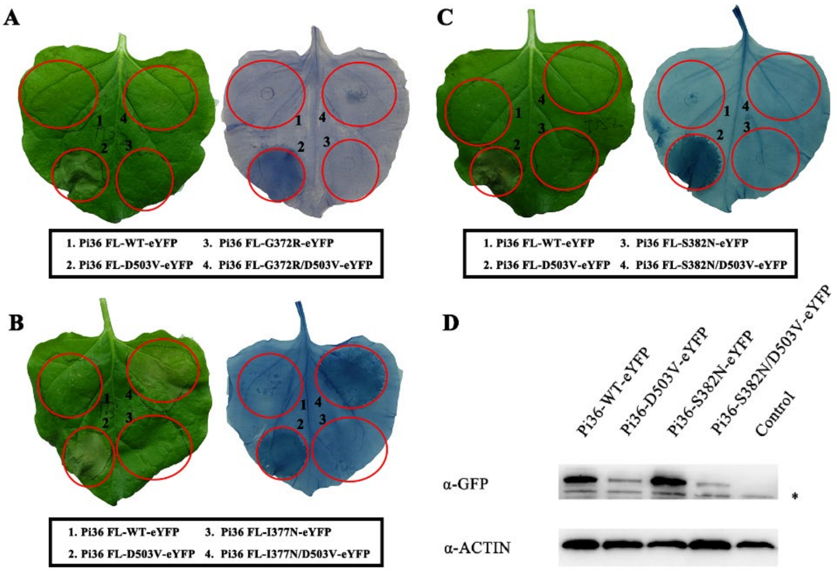

Figure S9. Analysis of cell death induction of Pi36 variants with mutations GLPL motif in *Nicotiana benthamiana*. (A-C) The cell death phenotype of Pi36 variants containing mutations in GLPL motif. The Pi36 variants with mutations were transiently expressed in *N.benthamiana* leaves to investigate the cell death activity. Representative leaves were photographed at 7dpi, followed by trypan blue staining. Three mutations in GLPL motifs could not autoactivated Pi36, but G372R and S382N abolished the cell death induced by Pi36-D503V. This experiment was repeated at least 3 times with similar results. (D) Protein expression level of Pi36 variants containing mutation S382N in GLPL motif. Total protein was extracted from agroinfiltrated leaves at 20hpi, and anti-GFP and anti-Actin antibodies were used to detect expression of fusion proteins. The asterisk indicates non-specific bands.

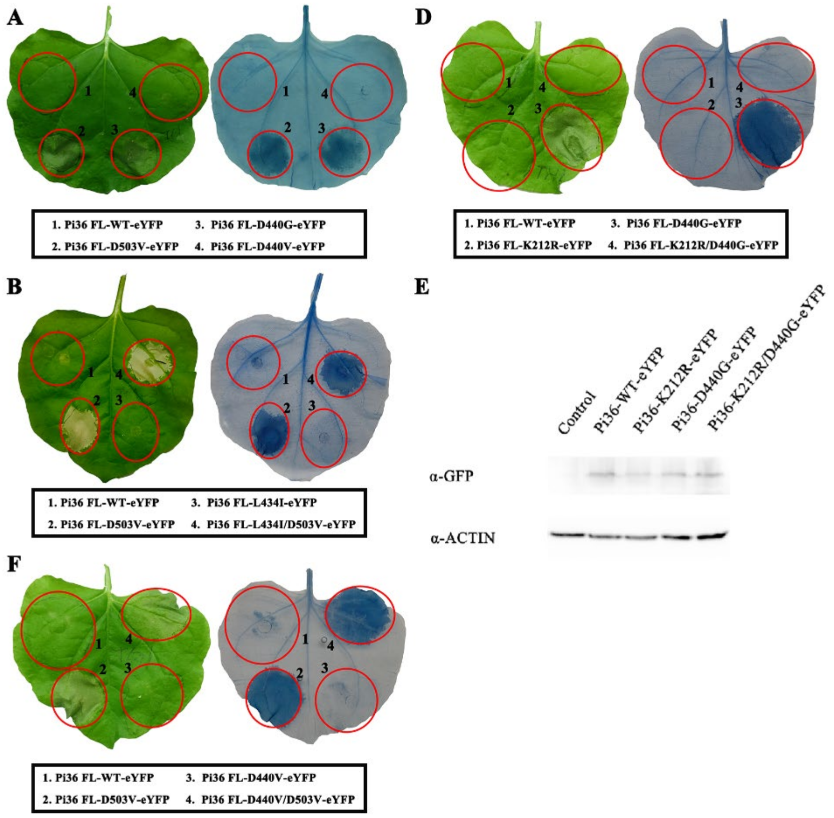

Figure S10. Analysis of cell death induction of Pi36 variants with mutations RNBS-D motif in *Nicotiana benthamiana*. (A-C) The cell death phenotype of Pi36 variants containing mutations in RNBS-D motif. Mutation D440G in RNBS-D motifs autoactivated Pi36. L434I and D440V could not autoactivate Pi36, but also not compromise cell death induced Pi36-D503V. (D-E) Pi36-D440G mediated cell death was compromised by mutation K212R in P-loop. The Pi36 variants with mutations were transiently expressed in *N.benthamiana* leaves to investigate the cell death activity. This experiment was repeated at least 3 times with similar results, and representative leaves were photographed at 7dpi, followed by trypan blue staining. For detection of fusion proteins expression, total proteins extracted from *N.benthamiana* leaves 20hpi were done by western blot with anti-GFP and anti-Actin antibodies.

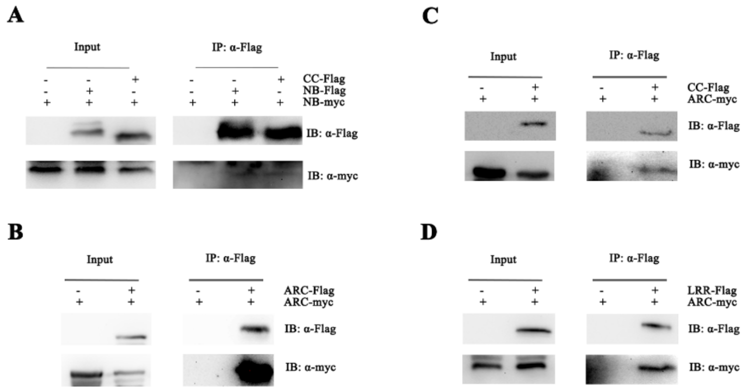

Figure S11. Interactions among Pi36 domains. (A-B) CC domain interacted with NB and ARC. (D) LRR domain also interacted with ARC. (A, C) Self-association of NB and ARC domain. Indicated combinations of Pi36 associated fusion proteins were transiently co-expressed in *N. benthamiana* for 20 hours. Total protein extracts were immunoprecipitated with anti-Flag beads, followed by immunoblot with anti-Flag and anti-Myc antibody.

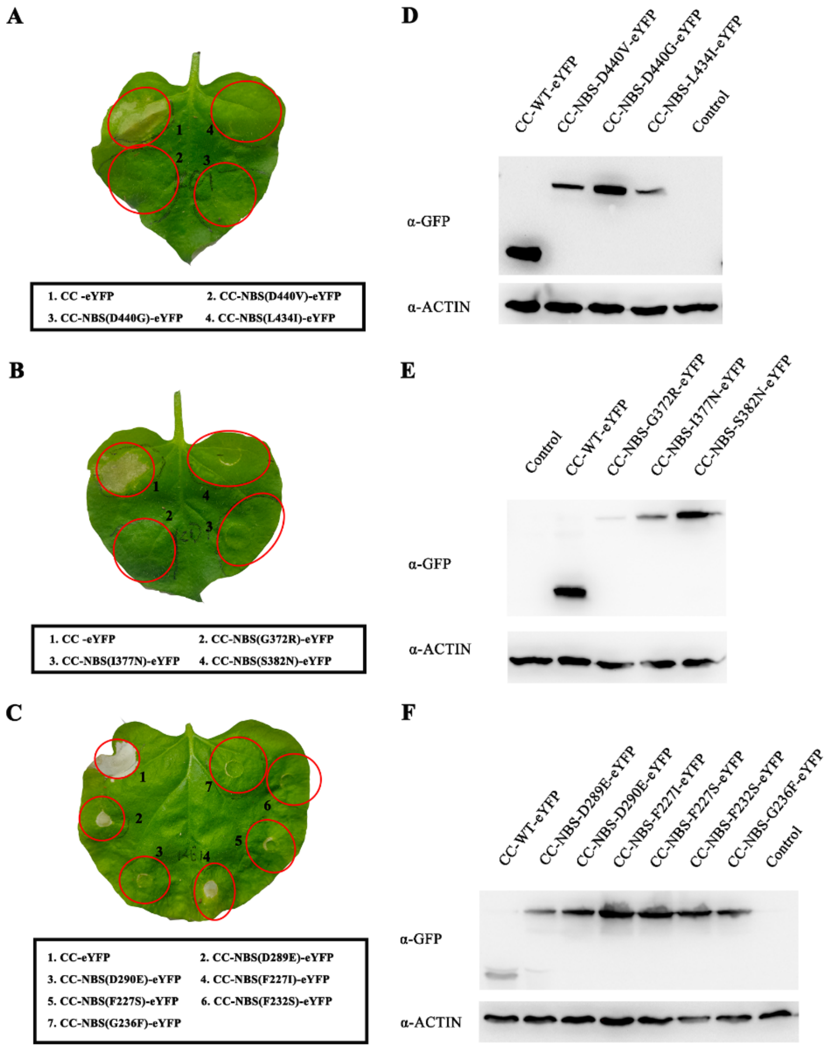

Figure S12. Analysis of cell death elicitation of additional CC-NB-ARC variants in *N.benthamiana*. (A-C) Cell death phenotypes induced by transient expression of Pi36 CC-NB-ARC variants in Nicotiana benthamiana. Pi36 CC-NB-ARC variants with mutations in conserved motifs fused to C-terminal eYFP tag, were transiently expressed in *N.benthamiana* by agroinfiltration. All expressed CC-NB-ARC variants could not induce invisible cell death phenotypes. This experiment was repeated at least 3 times with similar results, and photos were taken 7dpi. (D-F) Protein expression levels of each Pi36 variants. Total proteins were extracted from *N.benthamiana* leaves at 20hpi, and detection was conducted by western blot with anti-GFP and anti-Actin antibodies.

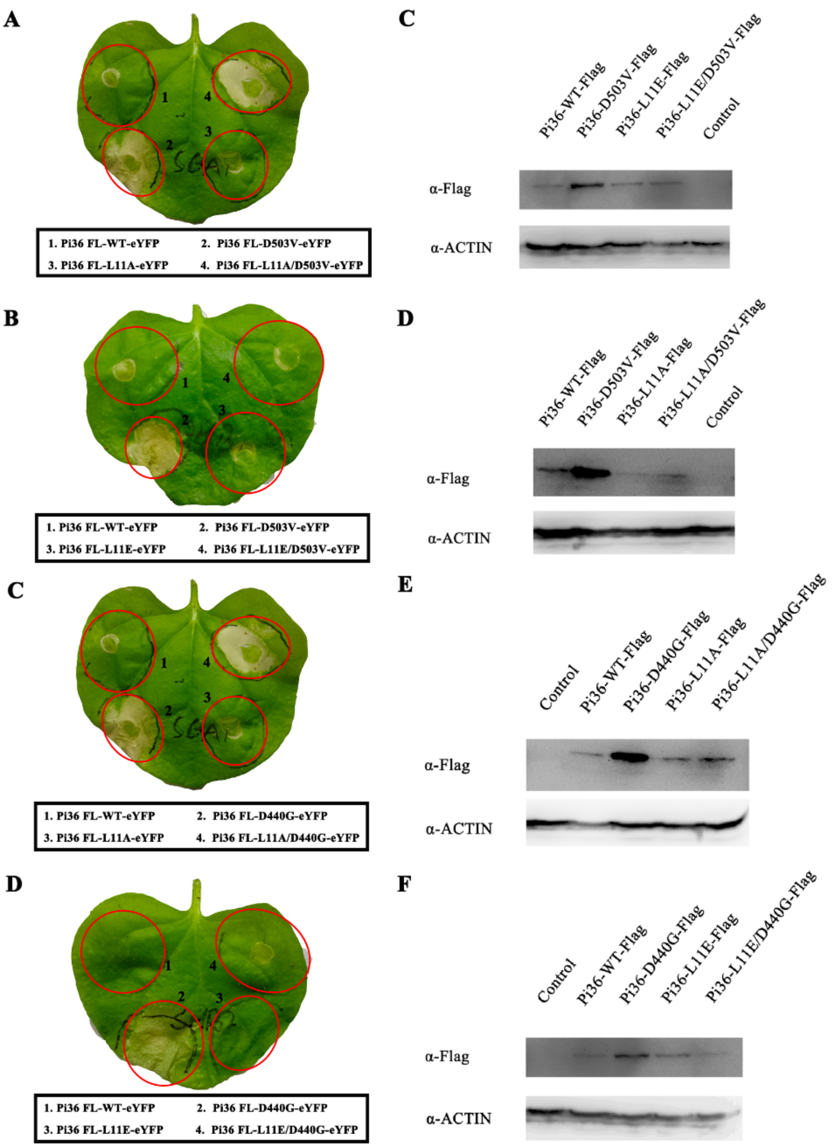

Figure S13. Investigating roles of residue L11 in Pi36 mediated cell death. (A-D) L11E compromised cell death induced by autoactive Pi36 variants in *N.benthamiana*. Two Pi36 autoactive variants Pi36-D503V and Pi36-D440G with mutations L11E or L11A in were fused to C-terminal eYFP tag, and Agrobacterium-mediated transient expression was conducted to assess the ability of cell death induction. Represented leaves were photographed at 7dpi. (D-F) Protein expression levels of each Pi36 variants. The expressed proteins were extracted from agro-infiltrated leaves collected at 20hpi, and detected by western blot with anti-GFP and anti-Actin antibodies.

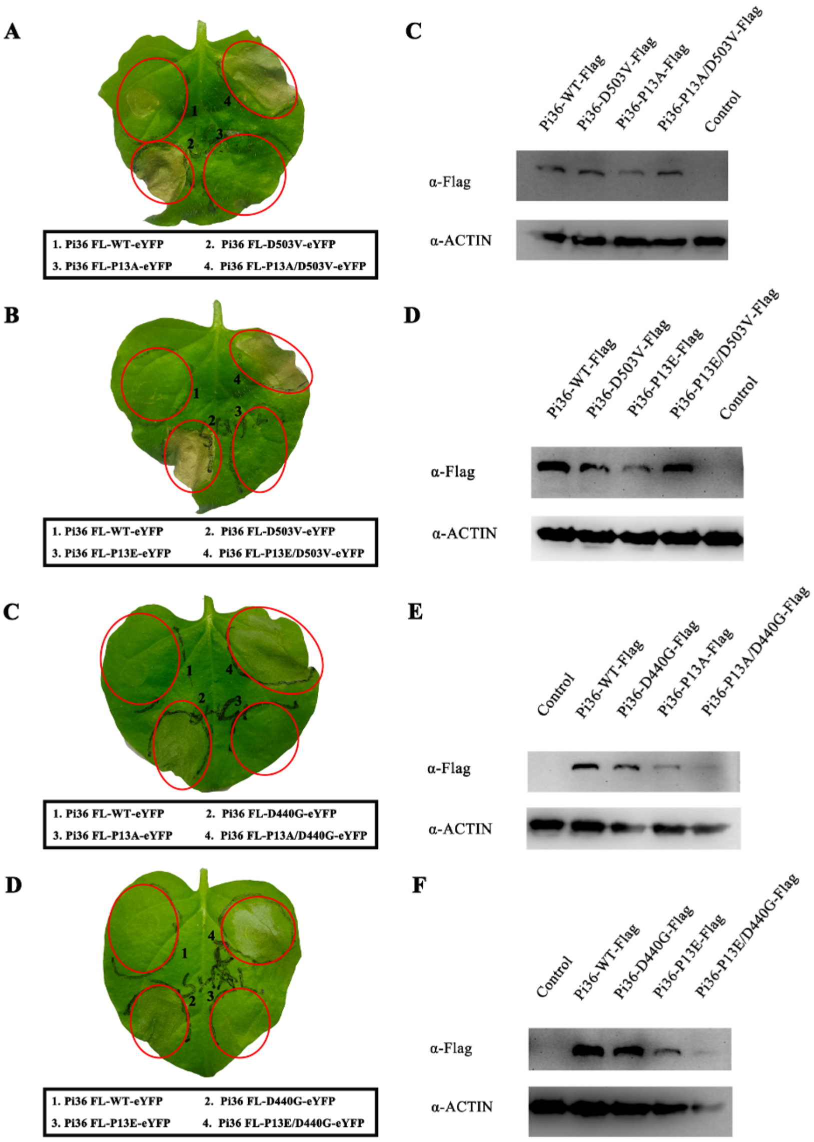

Figure S14. Investigating roles of residue P13 in Pi36 mediated cell death. (A-D) Mutations in site P13 could not compromise autoactive Pi36 mediated cell death. Pi36-D503V and Pi36-D440G with mutations P13A or P13E were fused to C-terminal eYFP tag, and transient expressed in *N.benthamiana* by agro-infiltration. The experiment was repeated at least 3 times, and represented leaves were photographed at 7dpi. (D-F) Protein expression levels of each Pi36 variants. Total proteins were extracted from agro-infiltrated *N.benthamiana* leaves at 20hpi, and detection was conducted by western blot with anti-GFP and anti-Actin antibodies.
